# Molecular Basis of Eusocial Complexity: The Case of Worker Reproductivity in Bees

**DOI:** 10.1093/gbe/evae269

**Published:** 2024-12-12

**Authors:** David C Prince, Anders Wirén, Timothy J Huggins, David H Collins, Tamas Dalmay, Andrew F G Bourke

**Affiliations:** School of Biological Sciences, University of East Anglia, Norwich Research Park, Norwich NR4 7TJ, UK; School of Biological Sciences, University of East Anglia, Norwich Research Park, Norwich NR4 7TJ, UK; School of Biological Sciences, University of East Anglia, Norwich Research Park, Norwich NR4 7TJ, UK; School of Biological Sciences, University of East Anglia, Norwich Research Park, Norwich NR4 7TJ, UK; School of Biological Sciences, University of East Anglia, Norwich Research Park, Norwich NR4 7TJ, UK; School of Biological Sciences, University of East Anglia, Norwich Research Park, Norwich NR4 7TJ, UK

**Keywords:** *Apis*, *Bombus*, gene expression, mRNA-seq, evolution of eusociality, worker reproduction

## Abstract

In eusocial insects, the molecular basis of worker reproductivity, including how it changes with eusocial complexity, remains relatively poorly understood. To address this, we used mRNA-seq to isolate genes differentially expressed between ovary-active and ovary-inactive workers in the intermediately eusocial bumblebee *Bombus terrestris*. By comparisons with data from the advanced eusocial honeybee *Apis mellifera*, which shows reduced worker reproductivity, we characterized gene expression differences associated with change in worker reproductivity as a function of eusocial complexity. By comparisons with genes associated with queen-worker caste development in *B. terrestris* larvae, we tested the behavioral–morphological caste homology hypothesis, which proposes co-option of genes influencing reproductive division of labor in adults in morphological caste evolution. We conducted comparisons having isolated genes expressed in *B. terrestris* worker-laid eggs to remove the potential confound caused by gene expression in eggs. Gene expression differences between the *B. terrestris* worker phenotypes were mainly in fat body and ovary, not brain. Many genes (86%) more highly expressed in ovary of ovary-active workers were also expressed in worker-laid eggs, confirming egg-expressed genes were potentially confounding. Comparisons across *B. terrestris* and *A. mellifera*, and with *B. terrestris* larvae, returned significant percentage overlaps in differentially expressed genes and/or enriched Gene Ontology terms, suggesting conserved gene functions underpin worker reproductivity as it declines with increasing eusocial complexity and providing support for the behavioral–morphological caste homology hypothesis. Therefore, within bees, both a degree of conserved gene use and gene co-option appear to underlie the molecular basis of worker reproductivity and morphological caste evolution.

**Significance**

In social insects, worker females have evolved to be decreasingly reproductive as social complexity has increased, but the molecular basis of this process remains little understood. Using RNA sequencing, we identified genes involved in worker reproduction in bumblebees, which show high levels of worker reproduction and an intermediate degree of social complexity. Comparisons first with honeybees, which show little worker reproduction and an advanced degree of social complexity, and then with bumblebee larvae, suggested that some shared genes continue to underpin worker reproductivity as social complexity falls, while others may become involved in larval development as adult queens or workers. Therefore both conserved gene use and gene co-option appear to contribute to the molecular basis of female reproductivity in bees.

## Introduction

The major transition to eusociality is characterized by a reproductive division of labor between reproductive phenotypes (queens or kings) and sterile or less reproductive phenotypes (workers) (Maynard Smith and Szathmáry 1995; Bourke 2011; Boomsma 2022). In obligate eusociality, adult workers are morphologically distinct from queens and generally unable to mate and thereby to found colonies independently (Boomsma 2022). At the ultimate (evolutionary) level, inclusive fitness theory explains this loss of reproduction in workers as kin-selected altruism of workers toward reproductive relatives (Hamilton 1964; West et al. 2007; Bourke 2011). However, at the proximate level, the genetic mechanisms underpinning the evolution of eusociality and associated traits remain relatively poorly understood. Recent molecular tools, including next-generation transcriptomics, mean that important inroads are being made into this central problem (Kapheim 2016; Toth and Rehan 2017; Favreau et al. 2018; Collins et al. 2021; Wyatt et al. 2023).

In the eusocial Hymenoptera (ants, bees, and wasps), unmated workers in many obligately eusocial species can produce haploid male offspring from unfertilized eggs via haplodiploidy (e.g. Bourke 1988). The degree to which workers vary in reproductive ability across eusocial lineages (the syndrome of traits characterized as “worker reproductivity”) is an important feature of eusociality because it profoundly affects the nature of reproductive conflicts within the colony (e.g. Ratnieks et al. 2006). In addition, eusocial species vary in their level of eusocial complexity, with relatively low queen-worker dimorphism and high worker reproductivity being associated with lower eusocial complexity (“primitive” or “intermediate” eusociality) and high queen-worker dimorphism and low worker reproductivity being associated with higher eusocial complexity (“advanced” eusociality) (Bourke 2011). Several studies have explored gene expression differences between reproductively active workers, reproductively inactive workers, and/or queens within obligately eusocial species (Pereboom et al. 2005; Grozinger et al. 2007; Cardoen et al. 2011; Harrison et al. 2015). However, few have sought to characterize such differences within workers in the context of variation in worker reproductivity occurring as a function of the degree of eusocial complexity.

Advanced eusociality is also associated with caste determination and differentiation (generating the morphological differences between adult queens and workers) occurring pre-imaginally, i.e. in larvae, and this is held to represent the derived state relative to reproductive division of labor being determined solely or mainly by behavioral differences among adults (Wheeler 1986). It has therefore been hypothesized that, during the evolution of eusociality, processes regulating reproductive division of labor in adults were co-opted to regulate caste determination and/or differentiation in larvae, establishing homology between these two sets of processes (Evans and Wheeler 1999). This hypothesis resembles the genetic toolkit hypothesis, which proposes that the evolution of complex social traits in divergent lineages has repeatedly co-opted a conserved set of genes and gene pathways in solitary ancestors (Amdam et al. 2004; Toth and Robinson 2007; Toth et al. 2010; Berens et al. 2014). However, to recognize its distinctness, we term the hypothesis that co-option of conserved genes has occurred in the change from behaviorally to morphologically defined castes the “behavioral–morphological caste homology (BMCH) hypothesis.” As such, this concept has been little tested (Evans and Wheeler 1999; Pereboom et al. 2005), especially using next-generation sequencing methods.

Comparisons between Bombini (bumblebees, *Bombus*) and Apini (honeybees, *Apis*) within the corbiculate bees provide a strong basis for investigating the molecular underpinnings of eusocial traits and their evolution. Bombini exhibits an “intermediate” level of eusociality (Amsalem et al. 2015a; Harrison et al. 2015; Zhuang et al. 2023), whereas Apini exhibits an “advanced” level. For example, although within both *Bombus* and *Apis* there are queen-worker differences in size, physiology, and behavior (Holland and Bloch 2020), *Bombus* queens and workers each have four ovarioles per ovary (Alford 1975; Duchateau and Velthuis 1989) whereas *Apis* queens and workers have, respectively, 150 to 180 ovarioles and 2 to 12 ovarioles per ovary (Winston 1987). In addition, the frequency of workers with fully activated ovaries and/or that lay eggs is much higher in mature colonies in *Bombus* than in *Apis* (*Bombus*: ∼40%; Amsalem et al. 2015a; *Apis*: 0.01%; Ratnieks 1993). (In queenless *Apis* colonies, this frequency is higher, up to 24% [Miller and Ratnieks 2001], but irreversible queenlessness is a short-lived and infrequent condition in *Apis*, since colonies can requeen themselves [only 1 of 25 colonies became irreversibly queenless in a study by Page and Metcalf (1984)], so worker reproductivity remains far lower than in *Bombus* when measured across the entire colony cycle.) Specifically, in the well-studied *B. terrestris*, a substantial fraction of workers lay eggs following the so-called “competition point” (date of first worker egg laying) (Duchateau and Velthuis 1989; Bloch and Hefetz 1999; Alaux et al. 2004; Zanette et al. 2012), and relatively high levels of worker reproductivity are widespread throughout the genus (Brown et al. 2003; Takahashi et al. 2010; Huth-Schwarz et al. 2011).

From phylogenetic evidence (Almeida et al. 2023), the common ancestor of *Bombus* and *Apis* is likely to have resembled an intermediately eusocial species. Therefore, although *Bombus* must have experienced independent social evolution since the split with the *Apis* lineage, *Apis* evolved far greater levels of queen-worker dimorphism and far lower levels of worker reproductivity following this split. Hence comparing the transcriptomes of reproductively active and inactive workers in each genus permits one to elucidate the molecular basis of worker reproductivity as the level of worker reproductivity decreases with increasing eusocial complexity. Similarly, comparisons within *Bombus* allow the BMCH hypothesis to be tested, because the genus exhibits behavioral reproductive division of labor within the worker caste and across queen and worker castes, as well as larval queen-worker caste determination (Amsalem et al. 2015a).

In the current study, we therefore conducted mRNA-seq on selected tissues to isolate genes differentially expressed between ovary-active and ovary-inactive *B. terrestris* workers and, via comparisons between *B. terrestris* and *A. mellifera* and within *B. terrestris*, to characterize gene expression differences associated with worker reproductivity as a function of the degree of eusocial complexity and to test the BMCH hypothesis. The selected tissues were brain, fat body, and ovary and were chosen because previous studies suggest that relevant pathways are localized in them (Grozinger et al. 2007; Page et al. 2012; Duncan et al. 2016; Lockett et al. 2016; Duncan et al. 2020). Overall, we sought to elucidate the molecular basis of worker reproductivity in an intermediately eusocial species and in the evolution of advanced from intermediate eusociality.

Several previous studies have investigated the genes differentially expressed between reproductive and non-reproductive workers in *Bombus* and *Apis* at the level of the transcriptome (e.g. *Bombus*: Pereboom et al. 2005 and Harrison et al. 2015; *Apis*: Grozinger et al. 2007, Cardoen et al. 2011, Galbraith et al. 2016, and Duncan et al. 2020) and of individual genes (e.g. *Bombus*: Amsalem et al. 2014, Lockett et al. 2016, and Amsalem et al. 2017; *Apis*: Duncan et al. 2016 and Ronai et al. 2016). Building on previous studies, we had three specific research goals. The first was to generate mRNA-seq-based gene expression profiles of ovary-active and ovary-inactive workers from the selected key tissues (brain, fat body, and ovary). For this, we also generated expression profiles for worker-laid eggs to remove, for the first time, the confound otherwise present in comparisons of gene expression profiles from ovary (or whole bodies in whole-body studies) in ovary-active versus ovary-inactive females. We therefore conducted comparisons involving ovary in ovary-active workers with egg-expressed genes (EEGs) excluded and included. Excluding EEGs conservatively assumes that these genes are not expressed in ovary tissue other than egg tissue, but avoids the potential mistake of inferring differential gene expression between ovary-active and ovary-inactive workers based solely on the presence of mature, unlaid eggs in ovary of ovary-active workers alone. Methodologically, we also sampled workers from a known colony context, age-matched workers across the phenotypes being compared (ovary-active vs. ovary-inactive workers) and phenotyped them (by ovarian dissection) using a standard scale.

The second research goal was to compare gene expression differences associated with worker reproductivity in *Bombus* and *Apis* to characterize such differences as a function of the degree of eusocial complexity by combining *B. terrestris* data from the current study with comparable published data from *A. mellifera*. For this, we determined the degree of overlap in genes differentially expressed, or in enriched gene pathways (Gene Ontology [GO] terms), between ovary-active and ovary-inactive workers across these two taxa.

The third research goal was to test the prediction of the BMCH hypothesis that genes and gene networks associated with adult female reproductivity overlap with those associated with caste determination and/or differentiation in larvae. In *B. terrestris*, the BMCH hypothesis predicts significant overlap of (i) genes (or GO terms) more highly differentially expressed (or enriched) in ovary-active workers (vs. ovary-inactive workers) and in mid to late instar queen-destined larvae (vs. worker-destined larvae) (BMCH hypothesis prediction 1) and (ii) genes (or GO terms) more highly differentially expressed (or enriched) in ovary-inactive workers (vs. ovary-active workers) and in mid to late instar worker-destined larvae (vs. queen-destined larvae) (BMCH hypothesis prediction 2). (We excluded early-instar larvae from these comparisons, as in *B. terrestris* early-instar larvae are totipotent, i.e. capable of following either caste pathway [Cnaani et al. 2000; Amsalem et al. 2015a].) Data on caste-associated genes in *B. terrestris* were taken from the mRNA-seq study of genes differentially expressed between phenotypically characterized and verified queen- and worker-destined *B. terrestris* larvae by Collins et al. (2021).

## Results

### mRNA-Seq: Overall Results

#### Brain, Fat Body, and Ovary Sequencing Experiment

Across the 54 libraries created for the 18 samples, mRNA-seq returned a mean of 33,805,603 reads per sample for brain, 34,186,153 reads per sample for fat body, and 33,919,435 reads per sample for ovary (supplementary table S1, Supplementary Material online). The libraries pseudoaligned to the *B. terrestris* transcriptome with a mean percentage pseudoalignment per sample of 76.1% (range 74.4% to 79.4%) for brain, 86.3% (86.1% to 86.6%) for fat body, and 78.7% (77.2% to 80.0%) for ovary (supplementary table S2, Supplementary Material online).

#### Egg Sequencing Experiment

Sequencing (by mRNA-seq) of the two libraries constructed from newly laid (≤1 h old) *B. terrestris* workers' eggs returned a mean of 66,890,753 reads per library (supplementary table S1, Supplementary Material online). The libraries pseudoaligned to the *B. terrestris* transcriptome with a mean percentage pseudoalignment of 76.6% (range 76.1% to 77.1%) (supplementary table S3, Supplementary Material online). Based on zFPKM analysis, a total of 7,828 genes were expressed in worker-laid eggs (Hart et al. 2013; Ammar and Thompson 2021) (supplementary table S4, Supplementary Material online).

#### Genes Differentially Expressed between Ovary-Active and Ovary-Inactive Workers of *B. terrestris*

**Fig. 1.**
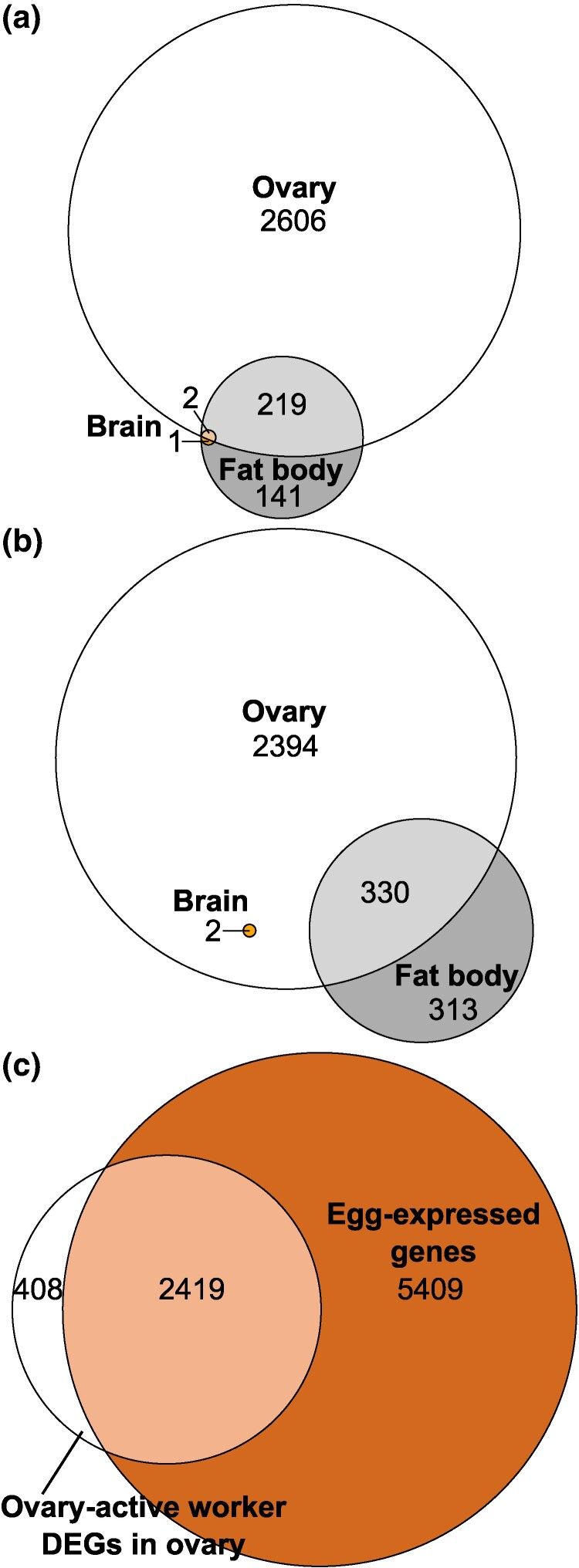
Comparison of gene expression profiles in *B. terrestris* worker tissues and eggs (current study). Euler diagrams of overlaps in DEGs from the mRNA-seq data, i.e. genes differentially expressed between ovary-active and ovary-inactive workers, between tissues, or in DEGs in ovary and EEGs. a) Ovary-active worker DEGs (genes more highly expressed in ovary-active workers) in brain, fat body, and ovary; b) ovary-inactive worker DEGs (genes more highly expressed in ovary-inactive workers) in brain, fat body, and ovary; c) ovary-active worker DEGs in ovary and genes expressed in worker-laid eggs. Numbers are number of genes in each category.

**Fig. 2.**
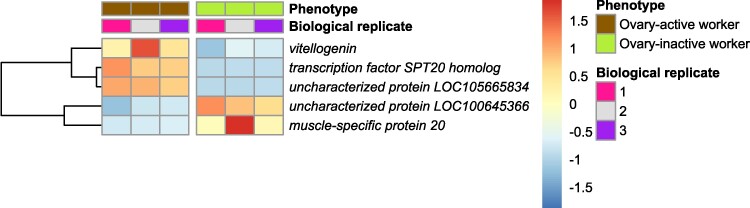
Gene expression in brain in ovary-active versus inactive *B. terrestris* workers (current study). Heatmap showing relative changes in gene expression (regularized log_2_-transformed counts) within each gene for all DEGs in brain (five in total), with each row representing an individual gene and each column representing a biological replicate from the mRNA-seq data. The vertical break separates samples from the two phenotypes (ovary-active and ovary-inactive workers). The dendrogram shows genes that cluster together according to their gene expression patterns.

**Fig. 3.**
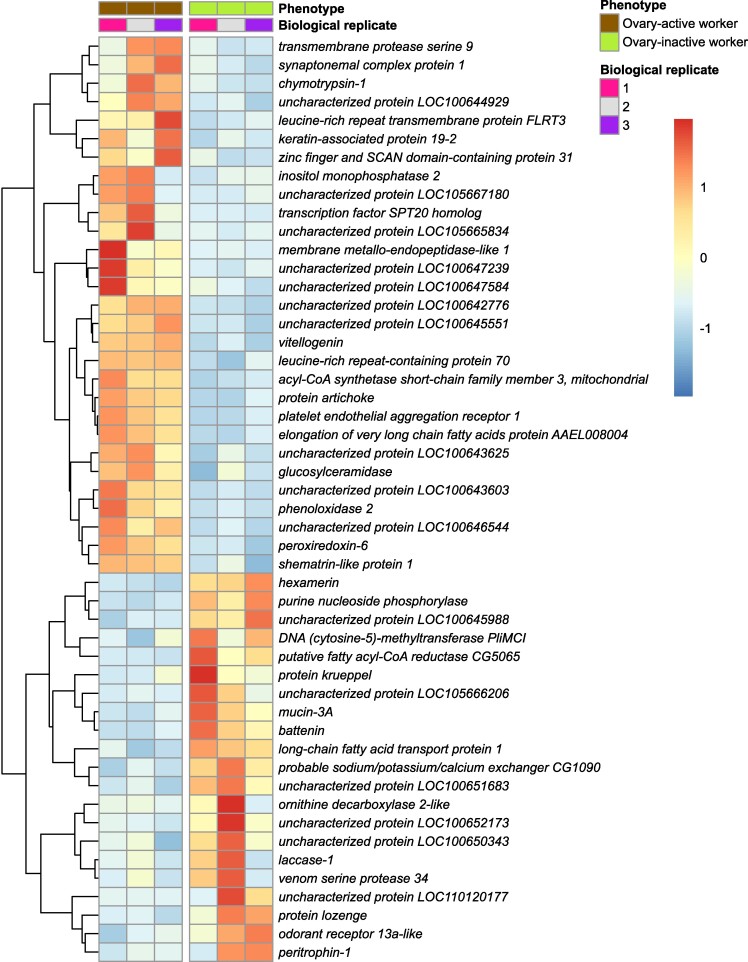
Gene expression in fat body in ovary-active versus inactive *B. terrestris* workers (current study). Heatmap showing relative changes in gene expression (regularized log_2_-transformed counts) within each gene for the 50 most DEGs in fat body (out of 1,006 DEGs in total), with each row representing an individual gene and each column representing a biological replicate from the mRNA-seq data. The vertical break separates samples from the two phenotypes (ovary-active and ovary-inactive workers). The dendrogram shows genes that cluster together according to their gene expression patterns.

**Fig. 4.**
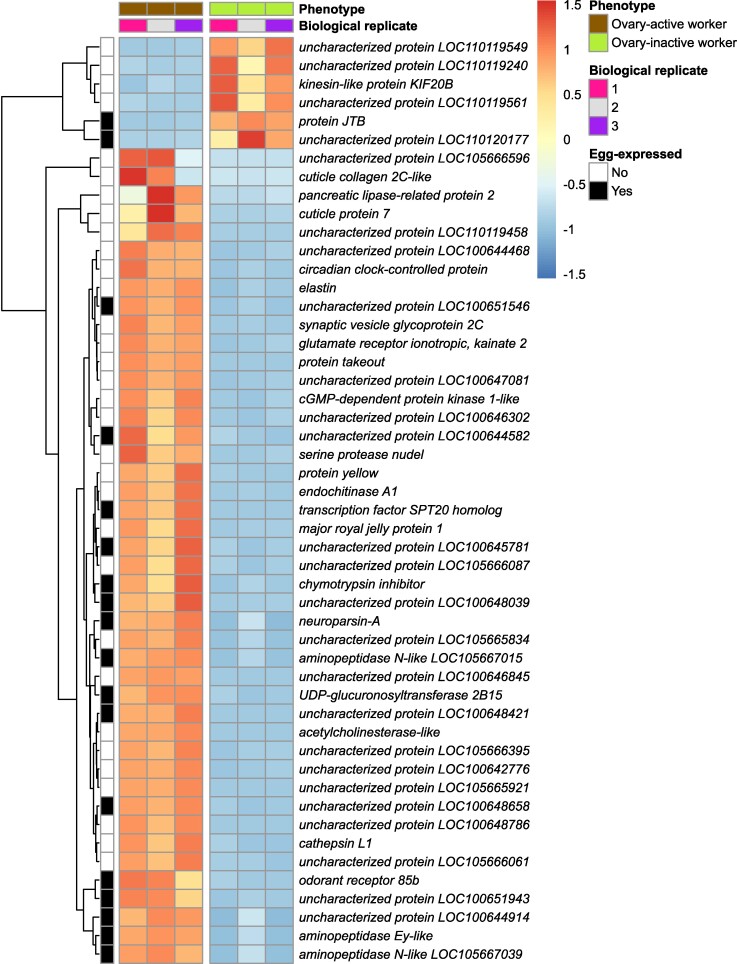
Gene expression in ovary in ovary-active versus inactive *B. terrestris* workers (current study). Heatmap showing relative changes in gene expression (regularized log_2_-transformed counts) within each gene for the 50 most DEGs in ovary (out of 5,553 DEGs in total), with each row representing an individual gene and each column representing a biological replicate from the mRNA-seq data. The vertical break separates samples from the two phenotypes (ovary-active and ovary-inactive workers). The dendrogram shows genes that cluster together according to their gene expression patterns (in ovary). The annotations at left represent whether the genes are expressed (yes—black) or not expressed (no—white) in worker-laid eggs.

In total, in both worker phenotypes (ovary-active and ovary-inactive workers) combined, there were 5 differentially expressed genes (DEGs) in brain, 1,006 DEGs in fat body, and 3,134 DEGs in ovary with EEGs excluded (5,553 DEGs in ovary with EEGs included) (Fig. 1). We defined DEGs more highly expressed in ovary-active than in ovary-inactive workers as “ovary-active worker DEGs” and DEGs more highly expressed in ovary-inactive than in ovary-active workers as “ovary-inactive worker DEGs.” In brain, there were three ovary-active worker DEGs and two ovary-inactive worker DEGs (Fig. 1a and b and 2; supplementary table S5, Supplementary Material online). In fat body, there were 363 ovary-active worker DEGs and 643 ovary-inactive worker DEGs (Fig. 1a and b and 3; supplementary table S6, Supplementary Material online). In ovary, there were 408 ovary-active worker DEGs with EEGs excluded (2,827 with EEGs included) and 2,726 ovary-inactive worker DEGs (Fig. 1a and b and 4; supplementary table S7, Supplementary Material online). Descriptively, the data showed that, among the ovary-active worker DEGs, one gene was differentially expressed in all three tissues (*uncharacterized protein LOC105665834*) with EEGs excluded (two genes with EEGs included, the additional gene being *transcription factor SPT20 homolog*), while 9% (31/363) of ovary-active worker DEGs in fat body were also differentially expressed in ovary of ovary-active workers with EEGs excluded (61% [221/363] with EEGs included) (Fig. 1a; supplementary table S8, Supplementary Material online). Among the ovary-inactive worker DEGs, no genes were differentially expressed in all three tissues, while two genes were differentially expressed in both brain and ovary (*uncharacterized protein LOC100645366* and *muscle-specific protein 20* [*Mp20*]) and 51% (330/643) of ovary-inactive worker DEGs in fat body were also differentially expressed in ovary of ovary-inactive workers (Fig. 1b; supplementary table S9, Supplementary Material online).

Of the 2,827 ovary-active worker DEGs in ovary, 86% were expressed in eggs (2,419 genes), leaving the 408 ovary-active worker DEGs in ovary with EEGs excluded (Table 1; Fig. 1c). However, of the ten most highly differentially expressed ovary-active worker DEGs in ovary, eight were not expressed in eggs and so remained among the ten most highly differentially expressed ovary-active worker DEGs in ovary when EEGs were excluded (supplementary table S7, Supplementary Material online).

**Table 1:** Gene expression in ovary-active workers and in haploid eggs in *Bombus* and *Apis*

Data source	Species	Tissue(s)	Total number of ovary-active worker DEGs	Number of ovary-active worker DEGs expressed in eggs	Number of ovary-active worker DEGs not expressed in eggs	Proportion (% of total DEGs) of ovary-active worker DEGs expressed in eggs
Current study	*B. terrestris*	Ovary	2,827	2,419	408	86%
Current study	*B. terrestris*	Combined fat body and ovary	2,674	2,321	353	87%
Harrison et al. (2015)	*B. terrestris*	Whole body	1,208	1,164	44	96%
Galbraith et al. (2016)	*A. mellifera*	Combined fat body and ovary	1,627	1,566	61	96%
Duncan et al. (2020)	*A. mellifera*	Ovary	2,785	2,725	60	98%

Numbers of DEGs in ovary-active workers of *B. terrestris* and *A. mellifera*, in newly laid eggs of workers (*B. terrestris*) (current study) or virgin queens (*A. mellifera*) (Pires et al. 2016) and in EEGs as a proportion of DEGs. Ovary-active worker DEGs, genes more highly expressed in ovary-active workers than in ovary-inactive workers.

Overall, of the three tissues investigated, ovary showed most differential gene expression, including when EEGs were excluded from ovary-active worker DEGs, followed by fat body, followed by brain (Fig. 1). The identities of the most highly DEGs in each tissue are detailed in Figs. 2 to 4 and supplementary tables S5 to S7, Supplementary Material online. The majority of differential gene expression (86% of DEGs) in ovary of ovary-active compared to ovary-inactive workers was attributable to gene expression in eggs within the ovaries of ovary-active workers. However, some differential gene expression remained between ovary tissue alone of the two worker phenotypes, including eight of the ten most highly differentially expressed DEGs (supplementary table S7, Supplementary Material online). These findings confirmed that genes expressed in eggs potentially confound comparisons of gene expression in ovary-active versus ovary-inactive females. Therefore, in ovary, subsequent analyses and comparisons focused on the data from ovary-active workers with EEGs excluded (results in the following sections). For the purposes of completeness and comparison, the results of analyses of data in ovary from ovary-active workers with EEGs included are available in supplementary results and figs. S1 to S4, Supplementary Material online.

#### GO Enrichment Analysis

Using OrthoFinder, we identified 6,025 single-copy orthologs between *B. terrestris* and *Drosophila melanogaster* (58.0% of the 10,383 genes expressed across the *B. terrestris* mRNA-seq libraries). We used these to isolate 264 non-redundant enriched GO terms for the DEGs (supplementary table S10, Supplementary Material online).

In brain, ovary-active worker DEGs were not enriched for GO terms, while ovary-inactive worker DEGs were enriched for terms associated with “syncytium” (3/11 non-redundant terms) (i.e. 3 non-redundant GO terms were associated with “syncytium” out of the total of 11 non-redundant GO terms enriched in brain ovary-inactive worker DEGs) and “myoblast/myotube” (3/11 non-redundant terms) (supplementary table S10, Supplementary Material online). In fat body, ovary-active worker DEGs were not enriched for GO terms, while ovary-inactive worker DEGs were enriched for terms associated with a range of processes including “regulation” (38/114 non-redundant terms) and “cell/cellular” (27/114 non-redundant terms) (supplementary table S10, Supplementary Material online). In ovary, ovary-active worker DEGs with EEGs excluded were enriched for “sensory perception” (GO:0007600) and “G-protein-couple receptor signaling pathway” (GO:0007186). Ovary-inactive worker DEGs in ovary were enriched for terms associated with a range of processes including “regulation” (41/114 non-redundant terms), “cell/cellular” (35/114 non-redundant terms), and “development” (16/114 non-redundant terms) (supplementary table S10, Supplementary Material online).

Comparison of DEGs and enriched GO terms between the current study and that of Harrison et al. (2015) showed that the two studies were broadly congruent (supplementary figs. S5 and S6 and tables S11 to S14, Supplementary Material online; for additional details, see supplementary results, Supplementary Material online).

### Comparison of Gene Expression Differences Associated with Worker Reproductivity in *Bombus* versus *Apis*

#### Comparison of DEGs between *B. terrestris* and *A. mellifera*

**Fig. 5.**
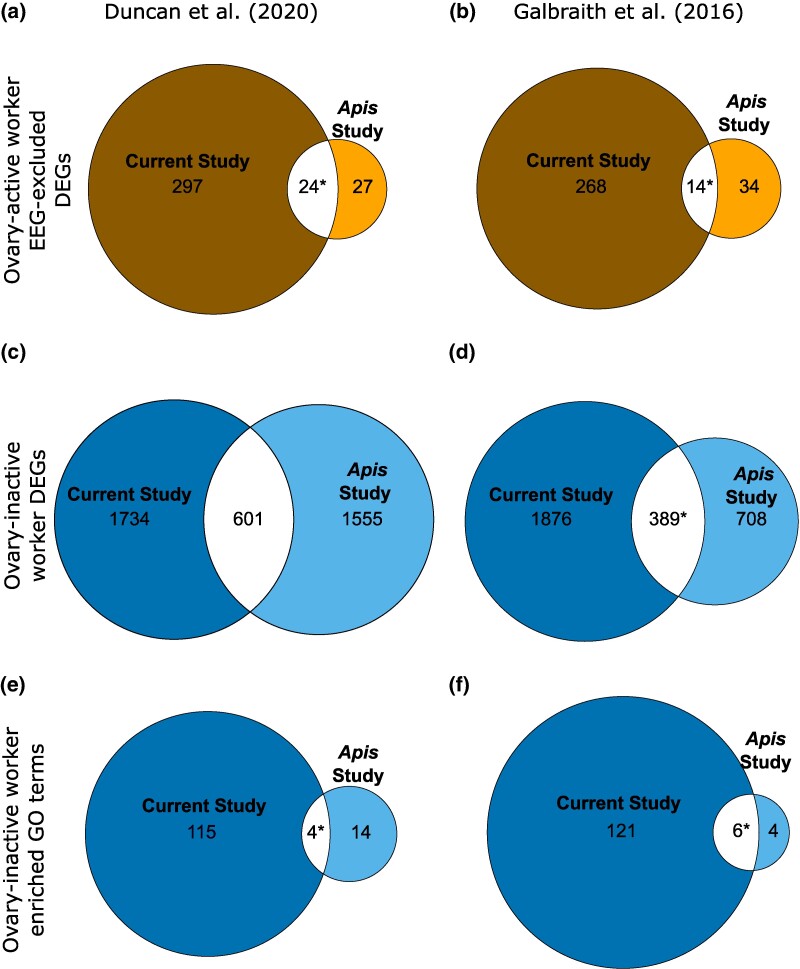
Comparison of gene expression and ontology in *B. terrestris* and *A. mellifera* workers. Euler diagrams of overlaps in DEGs or associated enriched GO terms from mRNA-seq data for ovary-active versus ovary-inactive workers between *B. terrestris* (current study) and *A. mellifera* (Duncan et al. 2020; Galbraith et al. 2016) (‘*Apis* study’) for the following combinations (where ovary-active worker DEGs are genes more highly expressed in ovary-active workers and ovary-inactive worker DEGs are genes more highly expressed in ovary-inactive workers): a) ovary-active worker DEGs in ovary with EEGs excluded and Duncan et al. (2020); b) ovary-active worker DEGs in combined fat body and ovary with EEGs excluded and Galbraith et al. (2016); c) ovary-inactive worker DEGs in ovary and Duncan et al. (2020); d) ovary-inactive worker DEGs in combined fat body and ovary and Galbraith et al. (2016); e) enriched GO terms from ovary-inactive worker DEGs in ovary and Duncan et al. (2020); f) enriched GO terms from ovary-inactive DEGs in combined fat body and ovary and Galbraith et al. (2016). Numbers are number of DEGs/enriched GO terms in each category. Asterisks (*), significant overlap in DEGs or enriched GO terms (Fisher's exact test, *P* < 0.05 after Bonferroni correction [adjusted *P* value threshold for significance = 0.017]). Results of statistical tests are in supplementary tables S15, S17, S20, and S22, Supplementary Material online, and identities of DEGs/enriched GO terms are in supplementary tables S16, S18, S21, and S23, Supplementary Material online.

Comparing ovary-active worker DEGs with EEGs excluded from *B. terrestris* ovary in the current study and *A. mellifera* ovary in Duncan et al. (2020) revealed significant overlap (7.5% of current study genes, *P* < 0.001; Fig. 5a; supplementary tables S15 and S16, Supplementary Material online).

Comparing ovary-active worker DEGs with EEGs excluded from *B. terrestris* combined fat body and ovary in the current study and *A. mellifera* combined fat body and ovary in Galbraith et al. (2016) also revealed significant overlap (5.0% of current study genes*, P* < 0.001; Fig. 5b; supplementary tables S17 and S18, Supplementary Material online). Comparing ovary-inactive worker DEGs between the current study and Duncan et al. (2020) revealed no significant overlap (25.7% of current study genes, *P* = 0.597; Fig. 5c; supplementary tables S15 and S16, Supplementary Material online), while comparing ovary-inactive worker DEGs between the current study and Galbraith et al. (2016) did reveal significant overlap (17.2% of current study genes, *P* < 0.001; Fig. 5d; supplementary tables S17 and S18, Supplementary Material online). For ovary-active worker DEGs with EEGs excluded and ovary-inactive worker DEGs, there were, respectively, 11 and 214 overlapping DEGs between the current study and both *A. mellifera* studies (supplementary table S19, Supplementary Material online). For ovary-active worker DEGs with EEGs excluded, the 11 overlapping DEGs included 3 *uncharacterized proteins,* as well as *protein takeout, major royal jelly protein 1* and *protein yellow*. Overall, therefore, *B. terrestris* and *A. mellifera* shared a significant percentage of DEGs in both (2/2) comparisons of ovary-active worker DEGs with EEGs excluded (albeit the percentage overlap was low, 5.0% to 7.5%) and a significant percentage of ovary-inactive worker DEGs in 1/2 comparisons (significant percentage overlap, 17.2%).

#### Comparison of Enriched GO Terms between *B. terrestris* and *A. mellifera*

Comparing enriched GO terms from ovary in the current study to enriched GO terms from *A. mellifera* ovary in Duncan et al. (2020) revealed significant overlap in GO terms enriched in ovary-inactive worker DEGs (3.4% of current study enriched GO terms, *P* = 0.001; Fig. 5e; supplementary tables S20 and S21, Supplementary Material online). Comparison between GO terms enriched in ovary-active worker DEGs with EEGs excluded was not possible, as no such enriched GO terms were found for Duncan et al. (2020). Comparing enriched GO terms from combined fat body and ovary in the current study to enriched GO terms from *A. mellifera* combined fat body and ovary in Galbraith et al. (2016) revealed significant overlap in GO terms enriched in ovary-inactive worker DEGs (4.7% of current study enriched GO terms, *P* < 0.001; Fig. 5f; supplementary tables S22 and S23, Supplementary Material online). Comparison between GO terms enriched in ovary-active worker DEGs with EEGs excluded was again not possible, as no such enriched GO terms were found for Galbraith et al. (2016). For ovary-inactive worker DEGs, two enriched GO terms were shared by all three studies: muscle structure development (GO:0061061) and myofibril assembly (GO:0030239). Overall, therefore, *B. terrestris* and *A. mellifera* shared a significant percentage of enriched GO terms in ovary-inactive worker DEGs in both (2/2) possible comparisons (albeit the percentage overlap was low, 3.4% to 4.7%).

### Behavioral–Morphological Caste Homology Hypothesis Tests

#### Comparison of DEGs between *B. terrestris* Workers and Larvae

**Fig. 6.**
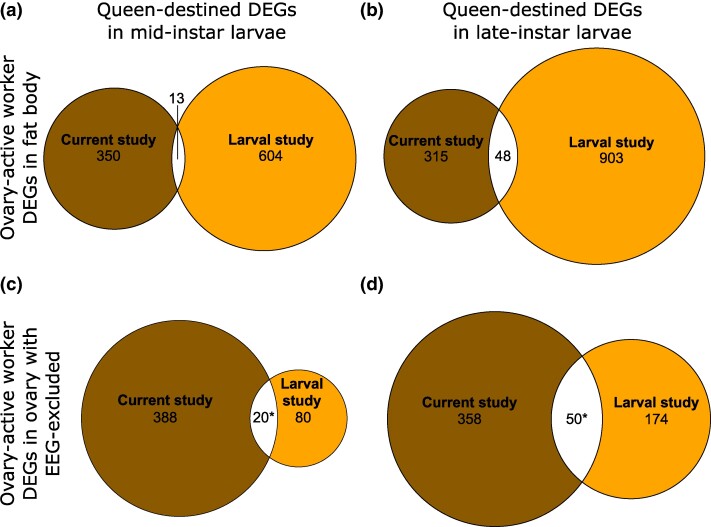
Comparison of gene expression in *B. terrestris* ovary-active adult workers and queen-destined larvae (BMCH hypothesis prediction 1). Euler diagrams of overlaps in DEGs from mRNA-seq data in fat body and ovary (with EEGs excluded) in ovary-active versus ovary-inactive *B. terrestris* workers (current study) and in mid and late-instars of queen-destined versus worker-destined *B. terrestris* whole larvae (Collins et al. 2021) (“Larval study”). a) Genes more highly expressed in fat body of ovary-active workers and mid-instar queen-destined larvae; b) genes more highly expressed in fat body of ovary-active workers and late-instar queen-destined larvae; c) genes more highly expressed in ovary of ovary-active workers and mid-instar queen-destined larvae (with EEGs removed); d) genes more highly expressed in ovary of ovary-active workers and late-instar queen-destined larvae (with EEGs removed). Numbers are number of DEGs in each category. Asterisks (*), significant overlap in DEGs (Fisher's exact test, *P* < 0.05 after Bonferroni correction [adjusted *P* value threshold for significance = 0.0083]). Results of statistical tests are in supplementary table S24, Supplementary Material online, and identities of DEGs are in supplementary table S25, Supplementary Material online.

**Fig. 7.**
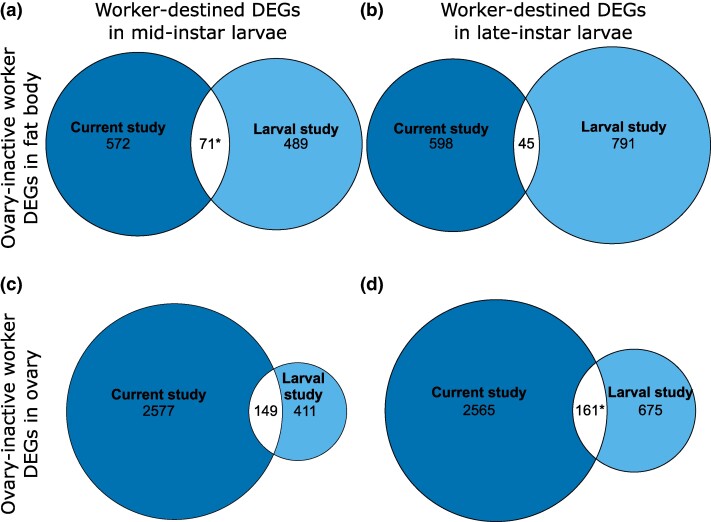
Comparison of gene expression in *B. terrestris* ovary-inactive adult workers and worker-destined larvae (BMCH hypothesis prediction 2). Euler diagrams of overlaps in DEGs from mRNA-seq data in fat body and ovary in ovary-active versus ovary-inactive *B. terrestris* workers (current study) and in mid and late-instars of queen-destined versus worker-destined *B. terrestris* whole larvae (Collins et al. 2021) (“Larval study”). a) Genes more highly expressed in fat body of ovary-inactive workers and mid-instar worker-destined larvae; b) genes more highly expressed in fat body of ovary-inactive workers and late-instar worker-destined larvae; c) genes more highly expressed in ovary of ovary-inactive workers and mid-instar worker-destined larvae; d) genes more highly expressed in ovary of ovary-inactive workers and late-instar worker-destined larvae. Numbers are number of DEGs in each category. Asterisks (*), significant overlap in DEGs (Fisher's exact test, *P* < 0.05 after Bonferroni correction [adjusted *P* value threshold for significance = 0.0083]). Results of statistical tests are in supplementary table S24, Supplementary Material online, and identities of DEGs are in supplementary table S25, Supplementary Material online.

As regards BMCH hypothesis prediction 1 at the gene level, the results showed no significant overlap between DEGs from fat body of ovary-active workers and either mid- or late-instar queen-destined larvae (Fig. 6a and b; supplementary tables S24 and S25, Supplementary Material online). They showed significant overlap between DEGs from ovary of ovary-active workers with EEGs excluded and queen-destined larvae for both mid and late instars (4.9% of current study genes, *P <* 0.001; Fig. 6c; 12.3% of current study genes, *P <* 0.001; Fig. 6d, respectively; supplementary tables S24 and S25, Supplementary Material online). Therefore, for prediction 1 at the gene level, 2/4 comparisons returned significant overlaps (range, 4.9% to 12.3%). For BMCH hypothesis prediction 2 at the gene level, the results showed significant overlap between DEGs from fat body of ovary-inactive workers and worker-destined larvae for mid (11.0% of current study genes, *P* < 0.001; Fig. 7a) but not late instars (Fig. 7b; supplementary tables S24 and S25, Supplementary Material online). They also showed no significant overlap between DEGs from ovary of ovary-inactive workers and worker-destined larvae at mid instars (Fig. 7c) but significant overlap at late instars (5.9% of current study genes, *P* < 0.001; Fig. 7d; supplementary tables S24 and S25, Supplementary Material online). Therefore, for prediction 2 at the gene level, 2/4 comparisons returned significant overlaps (range, 5.9% to 11.0%). The full list of overlapping genes is in supplementary table S25, Supplementary Material online.

#### Comparison of Enriched GO Terms between *B. terrestris* Workers and Larvae

Testing BMCH hypothesis prediction 1 at the GO level from either fat body or ovary data was not possible, as DEGs from fat body of ovary-active workers in the current study, and DEGs with EEGs excluded for mid and late instars of queen-destined larvae from Collins et al. (2021), were not enriched for any GO terms. For BMCH hypothesis prediction 2 at the GO level, comparing enriched GO terms derived from DEGs in the current study from either fat body or ovary of ovary-inactive workers and worker-destined larvae showed no significant overlap with either mid or late instars (0% of current study enriched GO terms, *P* = 1 in all cases) (supplementary figs. S7 and S8 and table S26, Supplementary Material online). For the prediction 2 tests, therefore, 4/4 possible comparisons returned no significant overlaps.

In summary, the results were consistent with BMCH hypothesis predictions 1 and 2 at the gene level (2/4 and 2/4 possible comparisons returning significant overlaps, respectively) but not at the level of enriched GO terms (4/4 possible comparisons returning no significant overlaps).

## Discussion

Using mRNA-seq, we isolated genes differentially expressed between age-matched ovary-active versus ovary-inactive *B. terrestris* workers in brain, fat body, and ovary. We also isolated genes expressed in *B. terrestris* worker-laid eggs, allowing us, for the first time, to make comparisons using genes differentially expressed in ovary without the potential confound caused by gene expression in eggs. By comparing our data with those from previous studies in *B. terrestris* and *A. mellifera*, we then characterized gene expression differences associated with worker reproductivity as a function of the degree of eusocial complexity in this lineage and tested the behavioral–morphological caste homology hypothesis. We now discuss the main findings as regards these goals.

### Differential Gene Expression and GO Differences between Ovary-Active and Ovary-Inactive Workers of *B. terrestris*

In the current study, we found the smallest number (5) of DEGs between the two worker phenotypes in brain (Fig. 1). By contrast, the mRNA-seq study of Marshall et al. (2019) found 334 DEGs in head tissue in ovary-active and ovary-inactive *B. terrestris* workers. The reasons for this difference are unknown but potentially stem from differences between the current study and Marshall et al. (2019) in tissue sampled (brain vs. head, respectively), social environment (queenright colonies [i.e. with a queen] vs. small queenless groups, respectively) and worker age (mean ∼28 d vs. 6 d, respectively). In *A. mellifera*, ovary-active workers differ in brain gene expression profile as a function of their level of egg-laying (Jones et al. 2020), and given our phenotyping of workers was based on ovary status alone, level of egg-laying could therefore have been an additional factor affecting DEG numbers in brain. The current study found 1,006 DEGs in fat body (Fig. 1), confirming that this tissue plays a key role in female reproductivity in bumblebees (Amsalem et al. 2015b; Lockett et al. 2016; Collins et al. 2023). We found the largest number of DEGs (3,134 with EEGs excluded), and the largest fold-changes in expression, in ovary (Fig. 1; supplementary tables S5 to S7, Supplementary Material online), consistent with the large morphological changes occurring in ovary during ovary activation in *B. terrestris* (Duchateau and Velthuis 1989). While the great majority (86%) of ovary-active worker DEGs in ovary were expressed in workers' eggs, suggesting that most differential gene expression in ovary between ovary-active versus inactive workers was influenced by gene expression in eggs, a number of DEGs (408) remained after EEGs were excluded (Table 1). These represent the set of genes conservatively estimated to be differentially more highly expressed in ovary of ovary-active compared to ovary-inactive workers. There was only one ovary-active worker DEG (*uncharacterized protein LOC105665834*) shared by all three tissues in the current study (there were two when EEGs were included, but the second, *transcription factor SPT20 homolog*, was also expressed in worker-laid eggs). *Uncharacterized protein LOC105665834* has homologs, as determined by *BLAST*, in other bee species; however, the potential function of the protein is unclear. While no ovary-inactive worker DEGs were shared by all three tissues in the current study, *uncharacterized protein LOC100645366* and *Mp20* were shared by brain and ovary. In *Drosophila*, the ortholog of *uncharacterized protein LOC100645366* is *dumpy* (*FBgn0053196*), which has diverse roles in extracellular matrix assembly, wing shape, and trachea development (Carmon et al. 2010a, 2010b), whereas *Mp20* is expressed predominantly in muscle cells (Vakaloglou et al. 2021).

Tian et al. (2021) showed using RNAi (RNA interference) that reduced expression of the gene *Immune Responsive Protein 30* (*IRP30*) decreased worker ovary-activation and egg-laying in *B. terrestris*. Similarly, Dong et al. (2020) found higher protein and mRNA levels for *IRP30* in egg-laying versus non-egg-laying workers in *Bombus lantschouensis*. Consistent with both these sets of findings, the current study showed that this gene (*LOC100642443*) was more highly expressed in ovary-active worker fat body and ovary and was not expressed in worker-laid eggs (supplementary tables S6 and S7, Supplementary Material online).

Our GO analyses found a variety of differences between the worker phenotypes in the three tissues. For example, in ovary, ovary-active worker DEGs with EEGs excluded were enriched for genes associated with the G-protein-couple receptor signaling pathway, suggesting that G-protein-couple receptors in ovary tissue itself may be important in ovary activation. In general, the DEGs and enriched GO terms from the current study, along with those from comparable previous studies (see Introduction), provide a basis for future functional studies of genes affecting worker reproductivity in bees.

### Comparison of Gene Expression Differences Associated with Worker Reproductivity in *Bombus* versus *Apis*

Comparison of *B. terrestris* worker DEGs from the current study and *A. mellifera* worker DEGs from two previous studies (Duncan et al. 2020; Galbraith et al. 2016) showed that *B. terrestris* and *A. mellifera* workers shared a significant percentage of ovary-active worker DEGs (with EEGs excluded) in both (2/2) comparisons and a significant percentage of ovary-inactive genes in 1/2 comparisons. While comparisons of enriched GO terms in ovary-active worker DEGs (with EEGs excluded) were not possible (because of a lack of enriched GO terms in the two *A. mellifera* studies when EEGs were excluded), the two species shared a significant percentage of enriched GO terms in ovary-inactive worker DEGs in 2/2 comparisons (Fig. 5). These results suggest that, within both *Bombus* and *Apis*, differences between workers in reproductivity are to some extent influenced by a shared set of conserved genes and pathways. Such conserved genes and pathways seem likely to be those involved, in individual workers, in shared processes of ovary activation downstream of those processes that potentially differ more between the lineages (see Introduction) and that affect such traits as the likelihood of becoming an ovary-active worker in the first place. For both DEGs and enriched GO terms, significant percentage overlaps were low (5.0% to 17.2% and 3.4% to 4.7%, respectively), so the proportion of conserved genes and pathways appears to be small. Therefore, the findings also suggest that, for the molecular underpinnings of worker reproductivity, and as also found for caste determination and/or differentiation (Collins et al. 2021), there has been a substantial level of independent evolution between the *Bombus* and *Apis* lineages. Similarly, when comparing across independent origins of eusociality, studies suggest roles for both conserved sets of genes and gene pathways and for novel genes (Berens et al. 2014; Mikheyev and Linksvayer 2015; Standage et al. 2016; Warner et al. 2019; Wyatt et al. 2023).

Eleven ovary-active worker DEGs were shared, after exclusion of EEGs, in all three studies (supplementary table S19, Supplementary Material online). Among these genes was *protein takeout*, which was more highly expressed in ovary of ovary-active workers in both *B. terrestris* and *A. mellifera*. There are currently no functional studies of the *D. melanogaster* ortholog (*Juvenile hormone binding protein 5* [*Jhbp5*]) (Vanaphan et al. 2012) of the *protein takeout* gene identified in the current study. The *A. mellifera* ortholog contains a predicted juvenile hormone (JH) binding domain (Hagai et al. 2007), and JH is a key regulator of ovary development and reproduction in female insects (Roy et al. 2018), including bumblebees (Amsalem et al. 2015a). However, in *A. mellifera*, JH appears mainly to regulate age-related division of labor, rather than reproduction (Pandey and Bloch 2015). Therefore, the exact, relative roles of this gene in *B. terrestris* and *A. mellifera* reproduction remain a subject for future investigation.

### Behavioral–Morphological Caste Homology Hypothesis Tests

Comparing DEGs associated with worker reproductivity (current study) with those associated with larval caste determination and/or differentiation (Collins et al. 2021) in *B. terrestris* yielded results for DEGs consistent, at least in part, with BMCH predictions 1 and 2 (2/4 and 2/4 comparisons returning significant overlaps, respectively). However, results for enriched GO terms supported neither prediction (for prediction 1, no comparisons were possible, and for prediction 2, 4/4 comparisons returned no significant overlaps). These findings provided support for the BMCH hypothesis with the qualification that, while some genes associated with the molecular underpinnings of adult worker reproductivity appear to have been co-opted for a role in queen-worker larval caste determination and/or differentiation, the genetic pathways affecting queen-worker larval caste determination and/or differentiation appear to have diverged from those affecting adult worker reproductivity. Percentages of DEGs overlapping were relatively small (range, 4.9% to 12.3% for prediction 1, 5.9% to 11.0% for prediction 2), reflecting that the numbers of shared genes were low (range, 20 to 140 for prediction 1, 45 to 161 for prediction 2). Nonetheless, the fact that both BMCH hypothesis predictions 1 and 2 were supported in part (at the gene level) by the DEG data suggests that, in *B. terrestris*, a small number of genes that promote adult worker reproduction have been co-opted to underpin caste determination and/or differentiation along the queen developmental pathway in larvae, while genes promoting adult worker sterility have been co-opted to underpin caste determination and/or differentiation along the worker developmental pathway in larvae. Future studies might therefore establish whether genes associated with adult queen-worker reproductive differences have been similarly co-opted in queen-worker larval caste determination and/or differentiation in *B. terrestris* and other eusocial taxa.

Among the genes with the largest fold-change that overlapped between ovary of ovary-active workers and late-instar queen-destined larvae, when EEGs were excluded (supplementary table S25, Supplementary Material online), was *major royal jelly protein 1 LOC100648898*. This gene was also more highly expressed in ovary-active workers compared to ovary-inactive workers (when EEGs were excluded) in both *B. terrestris* and *A. mellifera* (supplementary table S19, Supplementary Material online). While the *B. terrestris* annotation for the gene is *major royal jelly protein 1*, this is not the gene named *royal jelly protein like* (*RJPL*) investigated by Kupke et al. (2012) and Albert et al. (2014), which was *LOC100651683* (the homolog of *A. mellifera* ancestral *mrjp9* [Buttstedt et al. 2013]). Instead, the single-copy ortholog of *LOC100648898* (based on OrthoFinder analysis) in both *A. mellifera* (LOC413379) and *D. melanogaster* (FBgn0041709) is annotated as *yellow-g*. *Yellow-g* expression in ovaries of adults appears conserved in several insect species (Claycomb et al. 2004; Irles et al. 2009; Noh et al. 2020; Noh et al. 2023), while deficiency in *yellow-g* leads to abnormal eggs (Claycomb et al. 2004; Noh et al. 2020; Noh et al. 2023), which is consistent with increased *yellow-g* expression being linked with ovary activation and reproduction in *B. terrestris* and *A. mellifera* workers. Although, in *B. terrestris*, queen and workers share the same number of ovarioles per ovary (see Introduction), the higher expression of *yellow-g* in queen-destined versus worker-destined larvae suggests a possible molecular underpinning for the greater fecundity of queens.

### Conclusions

We isolated DEGs and enriched GO terms in brain, fat body, and ovary of ovary-active and ovary-inactive *B. terrestris* workers, as well as genes expressed in worker-laid eggs. The results showed that gene expression differences between the two worker phenotypes were mainly in fat body and ovary, with highly DEGs including *protein takeout* and *yellow-g*. In addition, 86% (2,419/2,827) of all ovary-active worker DEGs in ovary were expressed in worker-laid eggs (Table 1, Fig. 1). Similarly, excluding EEGs from ovary-active worker DEGs lists reduced gene list size by 96% in the *B. terrestris* data of Harrison et al. (2015) and 96% to 98% in the *A. mellifera* data of Duncan et al. (2020) and Galbraith et al. (2016) (Table 1). Therefore, removing these genes from ovary-active worker DEG lists can reduce the size of such lists by 86% to 98%. It follows that, unless excluded from whole-body, abdominal, or ovary gene lists, genes expressed in eggs potentially confound comparisons of gene expression in ovary-active versus ovary-inactive females (or in ovary-active females between species), as any differences or similarities in gene-expression profiles might mainly reflect presence or absence of eggs within samples. Excluding EEGs from ovary data tended to increase support for significant overlaps between genes and gene pathways associated with worker reproductivity in *Bombus* versus *Apis*, but such support remained whether EEGs were excluded or included (supplementary results and figs. S1 and S2, Supplementary Material online). BMCH hypothesis prediction 1 was supported at the gene level when EEGs were excluded, but not when they were included (supplementary results and figs. S3 and S4, Supplementary Material online). (The tests of BMCH hypothesis prediction 2 at the gene level were unaffected by excluding or including EEGs [supplementary results, Supplementary Material online] and returned overall positive results.) Because excluding EEGs resulted in the more conservative gene set, including EEGs in the case of prediction 1 at the gene level would therefore arguably have obscured important commonalities between differential expression of genes in ovarian tissue of adult ovary-active workers and in developing queen-destined larvae.

The comparisons of our *B. terrestris* data and the published *A. mellifera* data showed that it is likely that conserved gene functions played a role in the evolution of worker reproductivity in the change from intermediate to advanced eusociality in corbiculate bees and also that some independent evolution of gene function has occurred in this process, potentially associated with the far lower worker reproductivity in *Apis* relative to *Bombus*. Additional molecular phenomena not investigated in the current study might also be operative and include novel gene evolution (Sumner 2014), alternative splicing (Price et al. 2018), histone acetylation (Choppin et al. 2021), and DNA methylation (Amarasinghe et al. 2014; Li et al. 2018; Marshall et al. 2019). Comparisons of our data with lists of genes associated with larval queen-worker caste determination and/or differentiation in *B. terrestris* also provided support for the BMCH hypothesis at the gene level, suggesting that a number of genes influencing adult worker reproductivity have been co-opted to underpin queen-worker larval caste determination and/or differentiation within this lineage. Overall, therefore, our results suggest that, within bees, both a degree of conserved gene use and gene co-option underlie the molecular basis of worker reproductivity and caste determination and/or differentiation in the evolution of eusociality.

## Materials and Methods

### Bumblebee Colony Rearing

We obtained *Bombus terrestris audax* colonies, consisting of the queen, workers, and brood, from a commercial supplier, Biobest Belgium NV (Westerlo, Belgium) or Biobest UK Ltd (Ashford, UK) (see supplementary methods, Supplementary Material online). On receipt, we transferred colonies into individual wooden nest boxes with internal dimensions, 17 cm × 27.5 cm × 16 cm high, and counted the number of workers in each colony. We maintained all colonies in constant darkness at 28 °C and 60% humidity, conducting all manipulations under red light and supplying colonies ad libitum with sugar solution (“Biogluc,” Biobest Belgium NV/Biobest UK Ltd) and dried pollen (Koppert UK Ltd, Haverhill, UK).

### Sample Collection

#### Brain, Fat Body, and Ovary Sequencing Experiment

We removed workers for dissection from the queenright, post-competition point colonies when individually marked workers were 20 to 35 d old, with worker age defined as the number of days since eclosion (see supplementary methods, Supplementary Material online). We anesthetized each removed worker on ice for 10 min before dissection and then, to minimize RNA degradation, quickly dissected its brain, fat body, and ovaries on ice in bee Ringer's solution (Mercer and Menzel 1982) (see supplementary methods, Supplementary Material online). We scored each worker's level of ovary activation using a modification of the 6-point scale of Duchateau and Velthuis (1989). We defined “ovary-active workers” as those with ovaries scoring 4 to 6 (active ovaries) and “ovary-inactive workers” as those with ovaries scoring 0 to 1 (inactive ovaries) on the scale. We excluded workers scoring 2 to 3 (intermediate ovaries) from further analysis. We then stored dissected tissues in RNAlater (Sigma-Aldrich) in the case of brain and ovary and AllProtect (Qiagen, Manchester, UK) in the case of fat body at −20 °C until RNA extraction.

#### Egg Sequencing Experiment

To isolate genes expressed in eggs, we obtained expression profiles from *B. terrestris* worker-laid eggs sampled very shortly after being laid. Pires et al. (2016) found relatively few differences in gene expression in *A. mellifera* between mature oocytes and eggs aged less than 2 h (since being laid), thereby providing evidence that recently laid eggs represent a good proxy for mature oocytes. We established microcolonies each containing three *B. t. audax* workers of unknown age randomly selected from the same colony (see supplementary methods, Supplementary Material online). We housed each microcolony in a clear plastic box (7.5 cm × 14 cm × 5 cm high) with thawed wax from a different *B. t. audax* colony (Biobest UK Ltd) and provided sugar solution (“Attracker,” Koppert UK Ltd) and pollen (Biobest UK Ltd) ad libitum. We observed microcolonies for the presence of eggs. Once the first eggs had been laid, we removed all eggs in the microcolony and then removed any new eggs laid hourly (hence sampled eggs were up to 1 h old), flash froze them in liquid nitrogen within 5 min of collection, and stored them at −80 °C (see supplementary methods, Supplementary Material online).

### RNA Extraction, Library Construction, and Sequencing

#### Brain, Fat Body, and Ovary Sequencing Experiment

We pooled tissue samples to extract sufficient RNA for sequencing. For all three tissues in both phenotypes, we divided samples into three biological replicates. All replicates consisted of pooled tissues of 7 workers each, except for one replicate for brain of ovary-inactive workers that consisted of tissues of 14 workers (see supplementary methods, Supplementary Material online). In total, we created six samples per tissue (three for ovary-active workers and three for ovary-inactive workers). To control for worker age, we paired pools of ovary-active and ovary-inactive workers, matching pools within pairs as closely as possible by worker age while keeping the mean age of the workers in the pool similar. Age-matched workers were mostly from different colonies (21/21 and 18/21 pairs of age-matched workers for brain and fat body/ovary samples, respectively), as it was not possible to age-match ovary-active and ovary-inactive workers within a colony (supplementary table S27, Supplementary Material online). Following these procedures, brain samples for ovary-active workers comprised 3 biological replicates drawn, in total, from 21 individual workers from 6 colonies, with mean (range) age of workers of 28.3 (22 to 34) days. Brain samples for ovary-inactive workers comprised 3 biological replicates drawn, in total, from 28 individuals from 11 colonies, with mean (range) age of workers of 27.9 (24 to 32) days. Fat body and ovary samples for ovary-active workers each comprised 3 biological replicates drawn, in total, from 21 individual workers from 6 colonies, with mean (range) age of workers of 28.3 (22 to 34) days. Fat body and ovary samples for ovary-inactive workers each comprised 3 biological replicates drawn, in total, from 21 individual workers from 5 colonies, with mean (range) age of workers of 27.9 (24 to 32) days (for additional details, see supplementary methods, Supplementary Material online). We extracted total RNA from the samples, DNase-treated it, and assessed it for quality as described in supplementary methods, Supplementary Material online.

We sent samples to Edinburgh Genomics (Edinburgh, UK) for library construction and sequencing. The provider constructed a total of 18 TruSeq stranded mRNA libraries (Illumina, Cambridge, UK), i.e. from 2 phenotypes (ovary-active and ovary-inactive workers) × 3 tissues (brain, fat body, and ovary) each × 3 biological replicates (pooled samples) each, and sequenced the 18 libraries on each of three lanes of an Illumina HiSeq 2500, resulting in 54 raw data files (18 libraries × 3 technical replicates) of 50 base pair single-end reads.

#### Egg Sequencing Experiment

We created two samples of 24 eggs each from the worker-laid eggs, extracted total RNA from them, DNase-treated it, and then assessed it for quality as described in supplementary methods, Supplementary Material online. We sent these samples to BaseClear (Leiden, The Netherlands) for library construction and sequencing. The provider constructed two TruSeq stranded mRNA libraries (Illumina) and sequenced them on an Illumina HiSeq 2500, resulting in two raw data files of 50 base pair single-end reads.

### Bioinformatic Analysis

#### Brain, Fat Body, and Ovary Sequencing Experiment Analysis

We assessed the quality of the mRNA-seq reads using several complementary approaches. First, we used FastQC v0.11.9 (Andrews 2010) to examine a range of quality measures including base quality and potential adapter contamination in each sample, with the results for each sample combined into a report for each tissue (brain, fat body, and ovary) using the MultiQC v1.9 Python library (Ewels et al. 2016) with Python v3.7 (Python Core Team 2017) (supplementary files S1 to S3, Supplementary Material online). Subsequently, we aligned reads against the *B. terrestris* genome (Bombus_terrestris.Bter_1.0.dna.toplevel.fa) (Sadd et al. 2015) using HISAT2 v2.1.0 (Kim et al. 2015) and recorded mapping statistics (supplementary table S28, Supplementary Material online). We used the HISAT2 alignment files to assess gene body coverage and junction saturation using the RSeQC v3.0.1 Python library (Wang et al. 2012) with Python v3.7. We determined that each mRNA-seq library passed quality assessment and therefore retained all libraries for further analysis.

We pseudoaligned reads to the *B. terrestris* transcriptome (Bombus_terrestris.Bter_1.0.cdna.all.fa) with Kallisto v0.46.1 (Bray et al. 2016) (supplementary table S2, Supplementary Material online) and used the tximport package v1.22.0 (Soneson et al. 2016) in R (v4.1.3) (R Core Team 2018) to estimate transcript counts for each gene. We used these estimated counts for differential expression analysis in R (v4.1.3) (R Core Team 2018) with the DESeq2 package v1.34.0 (Love et al. 2014) using an FDR adjusted *P* value threshold of 0.05 and the model ∼ condition where condition was a categorical factor denoting worker ovary-activation status (“ovary-active” or “ovary-inactive”). We produced boxplots of the normalized count data and conducted principal component analysis (PCA) from DESeq2 for each tissue to check normalization and library clustering, respectively (supplementary figs. S9 to S11, Supplementary Material online). The subsequent PCA plots revealed that samples clustered by ovary-activation status of the worker in fat body and ovary (supplementary figs. S10 and S11, Supplementary Material online). In brain, one ovary-inactive worker library (OI_rep3) clustered with two of the ovary-active worker libraries (supplementary fig. S9, Supplementary Material online), but because it fell mid-way between the two other ovary-inactive worker libraries, it was retained in the analysis.

#### Egg Sequencing Experiment Analysis

Using the same workflow as described above (Brain, Fat Body, and Ovary Sequencing Experiment Analysis section) to assess the quality of the mRNA-seq reads (supplementary file S4 and table S29, Supplementary Material online), we determined that both libraries from worker-laid eggs passed quality assessment and therefore retained both for further analysis.

We pseudoaligned reads to the *B. terrestris* transcriptome (Bombus_terrestris.Bter_1.0.cdna.all.fa) with Kallisto v0.46.1 (Bray et al. 2016) and used the tximport package v1.22.0 (Soneson et al. 2016) in R (v4.1.3) (R Core Team 2018) to estimate transcript counts as “scaledTPM” for each gene. We transformed the counts using the zFPKM transformation (Hart et al. 2013) in the zFPKM package v1.16.0 (Ammar and Thompson 2021) in R (v4.1.3) (R Core Team 2018). Following Hart et al. (2013), we considered genes to be expressed if the zFPKM transformed estimate count value was greater than −3 in both libraries (supplementary table S4, Supplementary Material online). We then searched the earlier lists of ovary-active worker DEGs in ovary for the presence of genes expressed in worker-laid eggs and removed these genes to create the list of ovary-active worker DEGs in ovary with EEGs excluded.

#### GO Enrichment Analysis

To perform GO enrichment analysis, and comparative analyses with other gene lists, we used OrthoFinder v2.5.2 (Emms and Kelly 2019) to identify orthologs between *B. terrestris*, *D. melanogaster*, and *A. mellifera*. We used *D. melanogaster* single-copy orthologs for *B. terrestris* DEGs for GO enrichment analysis, as GO annotations for *D. melanogaster* are much more complete. We then performed GO enrichment analysis in R (v4.1.3) (R Core Team 2018) via the clusterProfiler package (v4.2.2) (Wu et al. 2021) using biological processes GO annotations from the org.Dm.eg.db package (v3.14.0) (Carlson 2021). We used an over-representation test (Boyle et al. 2004) to identify GO terms that were significantly over-represented (*P* < 0.05 after adjustment for multiple testing with *Benjamini-Hochberg*) in a set of DEGs against a background consisting of all genes that were expressed in the relevant tissue. We reduced redundancy in the resulting enriched GO terms using the GoSemSim package (v2.20.0) (Yu et al. 2010; Yu 2020).

### Comparison of DEGs and Enriched GO Terms between *B. terrestris* Studies

To assess congruence across studies, we compared DEGs and enriched GO terms from the current study with data from Harrison et al. (2015), who investigated gene expression in ovary-active versus ovary-inactive *B. terrestris* workers using a whole-body approach. To standardize comparisons, we reanalyzed the data from Harrison et al. (2015) with the bioinformatics pipeline used in the current study. We downloaded raw mRNA-seq files for Harrison et al. (2015) from the European Bioinformatics Institute (EBI) European Nucleotide Archive (ENA) (accession numbers: ERR883782-ERR883787) and determined DEGs and enriched GO terms as described above (Brain, Fat Body, and Ovary Sequencing Experiment Analysis and GO Enrichment Analysis sections) and in supplementary fig. S12 and tables S30 to S33, Supplementary Material online. As Harrison et al. (2015) used whole bodies (in pools) rather than specific tissues, we compared fat body and ovary data from the current study separately to their data. We conducted comparisons including and excluding EEGs and egg-associated GO terms from ovary-active worker DEGs and their enriched GO terms, respectively. We used two-tailed Fisher's exact tests to test for significant overlap between pairs of DEG lists or enriched GO term lists, with each test calculating whether the two lists being compared contained a greater-or-less-than-random number of shared items based on the respective sizes of the two lists and the total number of genes expressed in both data sets or GO terms derived from these expressed genes (in later Fisher's exact tests of the same design but applied between species, i.e. between *B. terrestris* and *A. mellifera*, the values corresponding to the latter values were the total number of shared orthologs expressed in both data sets or GO terms associated with the shared expressed orthologs).

### Comparison of Gene Expression Differences Associated with Worker Reproductivity in *Bombus* versus *Apis*

We searched the literature for previous mRNA-seq studies comparing gene expression between ovary-active and ovary-inactive workers in fat body or ovary in *A. mellifera* (omitting brain as the low number of DEGs found in brain in the current study prevented meaningful comparison [*Genes Differentially Expressed between Ovary-Active and Ovary-Inactive Workers of B. terrestris* section]). This search identified two studies suitable for the planned analyses, Duncan et al. (2020) (ovary data) and Galbraith et al. (2016) (combined fat body and ovary data). Workers in these two *Apis* studies came from queenright conditions (ovary-inactive workers, Duncan et al. (2020)) and queenless conditions (ovary-inactive workers, Galbraith et al. (2016); ovary-active workers, both studies). This was essentially because ovary-active workers occur only very rarely in queenright conditions in *Apis* (see Introduction). Therefore, given *Bombus* workers in our study were from queenright conditions, our *Bombus*/*Apis* comparisons assumed that, as in the original *Apis* studies, the main differences in ovary-active versus ovary-inactive worker gene expression remain comparable between queenright and queenless conditions.

#### DEGs

To standardize comparisons, we reanalyzed data from Duncan et al. (2020) and Galbraith et al. (2016) with the bioinformatics pipeline used in the current study. We downloaded raw mRNA-seq files for Duncan et al. (2020) and Galbraith et al. (2016) from the National Centre for Biotechnology Information (NCBI) gene expression omnibus (GEO) (accession numbers: GSE120563 and GSE76164, respectively) and determined DEGs as described above (Brain, Fat Body, and Ovary Sequencing Experiment Analysis section) and in supplementary figs. S13 and S14, tables S34 to S40, and file S5, Supplementary Material online. For the comparison with the data of Galbraith et al. (2016), which used fat body and ovary combined, we combined fat body and ovary mRNA-seq files from the current study and determined DEGs as described above (Brain, Fat Body, and Ovary Sequencing Experiment Analysis section) and in supplementary fig. S15 and tables S41 to S43, Supplementary Material online.

#### Determining Genes Expressed in *Apis mellifera* Haploid Eggs

As gene expression profiles for worker-laid *A. mellifera* eggs were not available, to exclude EEGs from the lists of DEGs in the *A. mellifera* studies of Duncan et al. (2020) and Galbraith et al. (2016), we analyzed mRNA-seq data from 0 to 2 h old *A. mellifera* eggs laid by virgin queens from Pires et al. (2016), since both virgin queens and workers lay unfertilized, haploid eggs. We downloaded these data from the NCBI Sequence Read Archive (SRA) (accession number: SRR1538449) and determined expressed genes as described above (Egg Sequencing Experiment Analysis section) and in supplementary tables S44 to S46, Supplementary Material online.

#### Comparison of DEGs and Enriched GO Terms between *B. terrestris* and *A. mellifera*

We used *D. melanogaster* single-copy orthologs for *A. mellifera* DEGs for GO enrichment analysis as described above (GO Enrichment Analysis section) and in supplementary tables S47 and S48, Supplementary Material online. We compared *B. terrestris* DEG and enriched GO term lists from ovary, or from combined fat body and ovary, from the current study with the corresponding lists of *A. mellifera* DEGs and enriched GO terms from Duncan et al. (2020) and Galbraith et al. (2016), respectively. We conducted both sets of comparisons excluding and including EEGs and egg-associated GO terms. We used Fisher's exact test to assess the degree of overlap between gene lists or enriched GO term lists as previously described (Comparison of DEGs and Enriched GO Terms between B. terrestris Studies section).

### Behavioral–Morphological Caste Homology Hypothesis Tests

To test BMCH hypothesis prediction 1, we compared DEGs and enriched GO terms between fat body and ovary in ovary-active workers (with EEGs either excluded or included) in the current study and queen-destined *B. terrestris* larvae (mid- and late-instar) in Collins et al. (2021), resulting, for each of the set of DEGs and GO terms, in two possible comparisons involving fat body, two involving ovary with EEGs excluded, and two involving ovary with EEGs included. Similarly, to test BMCH hypothesis prediction 2, we compared DEGs and enriched GO terms between fat body and ovary in ovary-inactive workers in the current study and worker-destined *B. terrestris* larvae (mid- and late-instar) in Collins et al. (2021), resulting, for each of the set of DEGs and GO terms, in two possible comparisons involving fat body and two involving ovary. For both predictions, we compared fat body and ovary separately to the larval data because Collins et al. (2021) used whole larvae (in pools) rather than specific tissues.

To standardize comparisons, we reanalyzed the mRNA-seq data from Collins et al. (2021) with the bioinformatics pipeline used in the current study. We downloaded raw mRNA-seq files for Collins et al. (2021) from NCBI GEO (accession number: GSE90751) and determined DEGs as described above (Brain, Fat Body, and Ovary Sequencing Experiment Analysis section) and in supplementary fig. S16 and tables S49 to S51, Supplementary Material online. We used *D. melanogaster* single-copy orthologs for *B. terrestris* DEGs for GO enrichment analysis as described above (GO Enrichment Analysis section) and in supplementary table S52, Supplementary Material online. We tested for overlaps in DEGs and enriched GO terms using Fisher's exact test as previously described (Comparison of DEGs and Enriched GO Terms between B. terrestris Studies section).

## Supplementary Material

evae269_Supplementary_Data

## Data Availability

All sequencing data generated by this article have been deposited in the National Center for Biotechnology Information's (NCBI's) Gene Expression Omnibus (GEO) at https://www.ncbi.nlm.nih.gov/geo/ and are available under series accession number GSE93274. The custom code used for the analyses is available at https://github.com/davidprince84/Prince-et-al_NE-L006758-1_exp1a_mRNA-seq_scripts.
