# Supplementary material for: Molecular Basis of Eusocial Complexity: The Case of Worker Reproductivity in Bees
Source: Genome Biol Evol. 2024 Dec 12;16(12):evae269. doi: 10.1093/gbe/evae269 (PMC11670783; doi:10.1093/gbe/evae269)
Supplement: evae269_Supplementary_Data [file evae269_supplementary_data.zip › GBE_ms_SM_revision_v6_2_no_track_changes.docx]

Supplementary Material for:

Molecular basis of eusocial complexity: the case of worker reproductivity in bees

David C. Prince*, Anders Wirén, Timothy J. Huggins, David H. Collins, Tamas Dalmay, Andrew F. G. Bourke

Corresponding Author: David C. Prince, [d.prince@uea.ac.uk](mailto:d.prince@uea.ac.uk)

**This file includes:**

Supplementary Methods

Supplementary Results

Supplementary References

Supplementary Figures S1 – S16

**Supplementary Methods**

**Bumble bee colony rearing**

For the brain, fat body and ovary sequencing experiment, we obtained 12 colonies (mean ± SD number of workers = 22 ± 9) from Biobest Belgium NV on 22 October 2014. To generate an additional pool of brain tissue, we obtained additional batches of 12 colonies (mean ± SD number of workers = 27 ± 12) and 15 colonies (mean ± SD number of workers = 16 ± 5) on 12 March 2015 and 2 April 2015, respectively, both from Biobest Belgium NV. For the egg sequencing experiment, we obtained three colonies from Biobest UK Ltd on 27 June 2016.

**Sample collection**

Brain, fat body and ovary sequencing experiment

We monitored colonies daily for callow (i.e. newly-eclosed) workers and all such workers were individually marked on the day they were detected with a numbered and coloured plastic disk (Swienty, Sønderborg, Denmark) glued to the thorax. Worker age was then defined as the number of days since marking, i.e. eclosion. We identified the competition point (date of first worker egg laying) for each colony by observing the colony every 1-2 days in two scans per day of three minutes per scan. Following Duchateau et al. (2004), a colony was considered to have passed the competition point when at least one of the following criteria was observed: 1) multiple open egg cells; 2) egg-eating by queen or workers; 3) aggression between queen and workers; or 4) egg-laying by workers. Once the competition point for a colony had been reached, we monitored workers daily in short observation bouts (5-10 min per day) to identify marked workers that were either a) seen to lay eggs or b) never seen to lay eggs. This was to screen for workers likely to be ovary-active or ovary-inactive, as confirmed by later dissection.

We conducted dissections under a stereomicroscope (Leica MZ6 microscope with Leica CLS 150X light source (Leica Microsystems, Milton Keynes, UK)) using a scalpel (No. 10, Swann Morton Limited, Sheffield, UK) and fine forceps (cleaned with RNAse Zap (Sigma-Aldrich, Gillingham, UK)). We dissected brains by removing the back of each worker's head with a scalpel, gently removing the brain with fine forceps, and then removing any attached glandular or muscle tissue. We dissected fat bodies by scraping from the inner wall of the abdominal cuticle while avoiding other tissues. We removed ovaries with fine forceps.

Egg sequencing experiment

To sample worker-laid eggs, we established microcolonies (n = 35) each containing three *B. t. audax* workers of unknown age randomly selected from the same colony (with each of three source colonies contributing, respectively, 12, 12 and 11 microcolonies in total). We placed eggs in batches in 2 ml microcentrifuge tubes (range of 4-9 eggs per batch), flash frozen in liquid nitrogen within 5 minutes of collection and stored at -80^o^C. We collected eggs from 12 of the 35 microcolonies over three different days (3, 4 and 5 August 2016) to yield a total of 85 worker-laid eggs each 0-1 h old (mean [range] number of eggs sampled per microcolony = 7.1 [3-10]) originating from all three source colonies.

**RNA extraction, library construction and sequencing**

Brain, fat body and ovary sequencing experiment

We conducted preliminary RNA extractions that suggested that 6-7 worker brains (the smallest of the three tissues used) was the minimum number needed to yield the 2.2 µg RNA recommended by the sequencing provider for mRNA-seq. Therefore, for all three tissues, we divided samples into pools each formed from tissues of 7 individual workers from 4 to 6 colonies per pool. This resulted in six pools, with pools one to three derived from ovary-active worker samples and pools four to six derived from ovary-inactive worker samples. In the case of pools four and five of brains of ovary-inactive workers, the RNA extracted was insufficient in quantity for library construction. We combined these two pools to form a new sample with sufficient RNA and a further brain sample was generated from 7 individual brains of ovary-inactive workers from 6 colonies obtained on 12 March 2015 and 2 April 2015 (pool 7). We paired pools by age with pool 1 being paired with pool 4, pool 2 being paired with pool 5 and pool 3 being paired with pool 6 in fat body and ovary. In brain, we paired pool 1 with pool 7, pool 2 was paired with pools 4 and 5 combined, and pool 3 was paired with pool 6. Details of ages, colonies and ovary-activation scores for each worker used for sequencing are available in Table S27.

To extract total RNA from a given tissue, we ground samples consisting of the pooled worker tissues in liquid nitrogen in 2 ml tubes using plastic 2 ml pestles (Eppendorf, Stevenage, UK), and suspended the powder in Tri-reagent (Ambion, Thermo Fisher Scientific, Loughborough, UK). We vortexed the samples and then centrifuged for 1.5 minutes. We removed supernatants to a fresh tube and, following the addition of an equal volume of 100% ethanol, we mixed the samples by vortexing. We bound the samples to the columns in the Direct-zol^TM^ RNA extraction kit (Zymo Research, Irvine, CA, USA). We followed the kit protocol, including a 15 minute on-column DNase treatment, finally eluting the column twice with the same 26 µl volume of DNase/RNase-free water. We carried out an additional DNase treatment using Turbo^TM^ DNase (Ambion) according to the manufacturer's protocol. We assessed RNA quantity and quality by agarose gel and Nanodrop 8000 (Thermo Fisher Scientific). The sequencing provider (Edinburgh Genomics) also assessed RNA quantity and quality with a 2200 TapeStation (Agilent, Cheadle, UK). RNA quantity (≥ 1.2 µg) and quality (A260/280 > 1.9, RIN ≥ 7.0 (RIN provided in TapeStation output)) were satisfactory for all samples except two of the initial brain samples from ovary-inactive workers, which did not contain sufficient RNA and hence were pooled as described above*.*

Egg sequencing experiment

We made two samples from the worker-laid eggs, each sample consisting of eight eggs from each of the three different collection days (i.e. 24 eggs per sample in total). We collected the 24 eggs for one sample from 6 different microcolonies originating from 3 source colonies, and the eggs for the other samples from 7 different microcolonies originating from 2 source colonies. We extracted RNA as described above (in 'Brain, fat body and ovary sequencing experiment'), finally eluting the Direct-zol^TM^ columns twice with the same 27 µl volume of DNase/RNase-free water. We assessed RNA quality by Nanodrop 8000 (Thermo Fisher Scientific), and the samples were found to have low 260/230 ratios. Therefore, we purified the RNA using the RNA Clean and Concentrator^TM^ -5 kit (Zymo Research), finally eluting the columns with 15 µl of DNase/RNase-free water. We carried out an additional DNase treatment using Turbo^TM^ DNase according to the manufacturer's protocol. We assessed RNA quantity and quality by agarose gel and Nanodrop 8000 (Thermo Fisher Scientific). The sequencing provider (BaseClear) assessed RNA quantity and quality with a Bioanalyzer 2100 (Agilent) and found the quality and quantity to be satisfactory for both samples (quantity >1.2 µg, A260/280 > 1.9, RIN not provided in Bioanalyzer output).

**Bioinformatic analysis**

Egg sequencing experiment analysis

We searched lists of ovary-active worker DEGs in ovary for the presence of genes expressed in worker-laid eggs and removed these genes to create the list of ovary-active worker DEGs in ovary with egg-expressed genes excluded (main text, 'Egg Sequencing Experiment Analysis'). However, we did not remove egg-expressed genes from lists of ovary-inactive worker DEGs in ovary. This was because, given ovary of ovary-inactive workers contains no eggs, these genes were more highly expressed in inactive ovaries than in active ovaries despite any expression in active ovaries that derived from the presence of eggs in the latter, and therefore were not potentially confounding.

**Comparison of DEGs and enriched GO terms between *B. terrestris* studies**

We used FastQC v0.11.9 (Andrews 2010) to examine a range of quality measures including base quality and potential adapter contamination in each sample from Harrison et al. (2015), with the results indicating that the sequencing files were of good quality (data not shown). We aligned reads from Harrison et al. (2015) against the *Bombus terrestris* genome (Bombus_terrestris.Bter_1.0.dna.toplevel.fa) (Sadd et al. 2015) using HISAT2 v2.1.0 (Kim et al. 2015) and recorded mapping statistics (Table S30).

We pseudoaligned reads from Harrison et al. (2015) to the *B. terrestris* transcriptome (Bombus_terrestris.Bter_1.0.cdna.all.fa) with Kallisto v0.46.1 (Bray et al. 2016) (Table S31) and used the tximport package v1.22.0 (Soneson et al. 2016) in R (v4.1.3) (R Core Team 2018) to estimate transcript counts for each gene. We used these estimated counts for differential expression analysis in R (v4.1.3) (R Core Team 2018) with the DESeq2 package v1.34.0 (Love et al. 2014) using an FDR adjusted *p-*value threshold of 0.05 and the model ~ condition where condition was a categorical factor denoting the ovary-activation status of the worker ('ovary-active' or 'ovary-inactive'). We produced boxplots of the normalised count data and principal component analysis (PCA) from DESeq2 for each tissue to check normalisation and library clustering, respectively (Figure S12). The subsequent PCA plot revealed that samples clustered by ovary-activation status of the worker, except library OI_C09, which clustered with ovary-active worker libraries rather than ovary-inactive worker libraries (as was noted by Harrison et al. (2015)). Therefore, library OI_C09 was excluded from further analysis and the differential expression analysis for Harrison et al. (2015) repeated, and a new boxplot and PCA produced (Figure S12C, D). Details of gene expression, including differentially expressed genes, can be found in Table S32.

We performed Gene ontology (GO) enrichment analysis for the DEGs from Harrison et al. (2015) as described for DEGs from the current study in the main text, 'Materials and Methods, GO Enrichment Analysis'. Details of the GO terms shown to be enriched can be found in Table S33.

**Comparison of gene expression differences associated with worker reproductivity in *Bombus* vs. *Apis***

Differentially expressed genes

Details of how ovary data from the current study were analyzed are available in the main text (Brain, Fat Body, and Ovary Sequencing Experiment Analysis). We used FastQC v0.11.9 (Andrews 2015) to examine a range of quality measures including base quality and potential adapter contamination in each sample from Duncan et al. (2020), Galbraith et al. (2016) and combined fat body and ovary from the current study. The FastQC results from Duncan et al. (2020) and combined fat body and ovary from the current study indicated that the sequencing files were of good quality (data not shown). However, the FastQC results for Galbraith et al. (2016) (Supplementary file S5) indicated several concerns: all of the files containing read 2 of the paired reads experienced a decrease in quality between position 54 and 60 in the read; this correlated with a large increase in the percentage of bases called as N (i.e. the base could not be accurately determined) in this position; and many files included an increased number of over-represented sequences. We aligned reads from Duncan et al. (2020) and Galbraith et al. (2016) against the *Apis mellifera* genome (Apis_mellifera.Amel_HAv3.1.dna.toplevel.fa.gz) (Wallberg et al. 2019) and reads from combined fat body and ovary from the current study against the *Bombus terrestris* genome (Bombus_terrestris.Bter_1.0.dna.toplevel.fa) (Sadd et al. 2015) using HISAT2 v2.1.0 (Kim et al. 2015) and recorded mapping statistics (Tables S34, S35 and S41). Very few paired reads from Galbraith et al. (2016) mapped uniquely (less than 1% of any library, Table S35), therefore all files containing read 1 of the pairs were mapped as single-end reads (due to the quality issue identified by FastQC in the read 2 files). This led to good overall alignment rates (>80%) in all but three samples: OA_8820, OA_882c and OI_882k (Table S36). However, most samples contained a high proportion of multi-mapping reads (>15%, compared to a maximum in Duncan et al. (2020) of ~5%, Tables S34, S36).

We pseudoaligned reads from Duncan et al. (2020) and read 1 of the pairs as single-end reads from Galbraith et al. (2016) against the *A. mellifera* transcriptome (Apis_mellifera.Amel_HAv3.1.cdna.all.fa.gz) and reads from combined fat body and ovary from the current study to the *B. terrestris* transcriptome (Bombus_terrestris.Bter_1.0.cdna.all.fa) with Kallisto v0.46.1 (Bray et al. 2016) (Tables S37, S38 and S42) and used the tximport package v1.22.0 (Soneson et al. 2016) in R (v4.1.3) (R Core Team 2018) to estimate transcript counts for each gene. We used these estimated counts for differential expression analysis in R (v4.1.3) (R Core Team 2018) with the DESeq2 package v1.34.0 (Love et al. 2014) using an FDR adjusted p-value threshold of 0.05 and the model ~ condition where condition was a categorical factor denoting the ovary-activation status of the worker ('ovary-active' or 'ovary-inactive'). We produced boxplots of the normalised count data and principal component analysis (PCA) from DESeq2 for each tissue to check normalisation and library clustering, respectively (Figures S13 – S15). The subsequent PCA plots revealed that samples in Duncan et al. (2020) and combined fat body and ovary clustered by phenotype, and therefore no samples were excluded from further analysis. The PCA plot for Galbraith et al. (2016) revealed four samples that clustered with samples from the opposite phenotype (OI_875a, OI_888u, OA_894r and OA_894q). Analysis of the HISAT2 alignment and Kallisto pseudoalignment results (Tables S13, S15) identified four further samples with a HISAT2 overall alignment rate less than 90% and/or a Kallisto pseudoalignment rate less than 70% (OA_8820, OA_882c, OI_882k and OA_8754). Therefore, these eight samples (OI_875a, OI_888u, OA_894r, OA_894q, OA_8820, OA_882c, OI_882k and OA_8754) were excluded from further analysis and the differential expression analysis for Galbraith et al. (2016) repeated, and a new boxplot and PCA produced (Figure S14C, D). Details of gene expression, including differentially expressed genes, can be found for Duncan et al. (2020), Galbraith et al. (2016) and combined fat body and ovary from the current study in Tables S39, S40 and S43, respectively.

Determining genes expressed in *Apis mellifera* haploid eggs

We aligned reads from Pires et al. (2016) against the *Apis mellifera* genome (Apis_mellifera.Amel_HAv3.1.dna.toplevel.fa.gz) (Wallberg et al. 2019) using HISAT2 v2.1.0 (Kim et al. 2015) and recorded mapping statistics (Table S44).

We pseudoaligned reads from Pires et al. (2016) against the *A. mellifera* transcriptome (Apis_mellifera.Amel_HAv3.1.cdna.all.fa.gz) with Kallisto v0.46.1 (Bray et al. 2016) (Table S22) and used the tximport package v1.22.0 (Soneson et al. 2016) in R (v4.1.3) (R Core Team 2018) to estimate transcript counts as 'scaledTPM' for each gene. The counts were transformed using the zFPKM transformation (Hart et al. 2013) in the zFPKM package v1.16.0 (Ammar and Thompson 2021) in R (v4.1.3) (R Core Team 2018). As recommended by Hart et al. (2013), genes in haploid eggs were considered expressed if the zFPKM transformed estimate count value was greater than -3 (Table S46). The lists of differentially expressed genes (DEGs) more expressed in ovary of ovary-active workers from Duncan et al. (2020) and/or combined fat body and ovary of ovary-active workers from Galbraith et al. (2016) were then searched for the presence of genes expressed in *A. mellifera* haploid eggs.

**Behavioural-morphological caste homology hypothesis tests**

Differentially expressed genes

We aligned reads from Collins et al. (2021) against the *Bombus terrestris* genome (Bombus_terrestris.Bter_1.0.dna.toplevel.fa) (Sadd et al. 2015) using HISAT2 v2.1.0 (Kim et al. 2015) and recorded mapping statistics (Table S49).

We pseudoaligned reads from Collins et al. (2021) to the *B. terrestris* transcriptome (Bombus_terrestris.Bter_1.0.cdna.all.fa) with Kallisto v0.46.1 (Bray et al. 2016) (Table S50) and used the tximport package v1.22.0 (Soneson et al. 2016) in R (v4.1.3) (R Core Team 2018) to estimate transcript counts for each gene. We used these estimated counts for differential expression analysis in R (v4.1.3) (R Core Team 2018) with the DESeq2 package v1.34.0 (Love et al. 2014) using an FDR adjusted p-value threshold of 0.05 and the model ~ condition where condition was a categorical factor denoting the instar ('early', 'mid' or 'late') and caste ('queen-destined' or 'worker-destined') of the larvae. (We performed analyses on the early-instar larvae for completeness but report them only in Table S51, because, as described in the main text, *B. terrestris* early-instar larvae are totipotent, i.e. capable of following either a queen- or worker-destined caste pathway.) We produced boxplots of the normalised count data and principal component analysis (PCA) from DESeq2 for each tissue to check normalisation and library clustering, respectively (Figures S16). The subsequent PCA plots revealed that samples clustered by instar stage, and therefore all libraries were retained for further analysis. Details of gene expression, including differentially expressed genes, can be found in Table S51.

**Supplementary Results**

**Gene ontology (GO) enrichment analysis in *B. terrestris* for ovary-active worker DEGs with egg-expressed genes included**

In ovary, ovary-active worker DEGs with egg-expressed genes included were enriched for GO terms associated with 'protein localization/transport' (4/13 non-redundant terms) and 'metabolic process' (3/13 terms) (Table S10).

**Comparison of DEGs and enriched GO terms between *B. terrestris* studies**

The list of ovary-active worker DEGs in fat body from the current study did not significantly overlap (13.8% of current study genes, *p* = 0.468) with the list of ovary-active worker DEGs in whole body samples from Harrison et al. (2015), while the lists of ovary-inactive worker DEGs did significantly overlap (29.4% of current study genes, *p* < 0.001) across the two studies (Fig. S5, Tables S11, S12). The lists of both ovary-active and ovary-inactive worker DEGs in ovary from the current study significantly overlapped (26.2% of current study genes, *p* < 0.001; 17.8% of current study genes, *p* < 0.001, respectively) with the corresponding whole-body lists from Harrison et al. (2015) (Fig. S5, Tables S11, S12). Excluding egg-expressed genes from each study's ovary-active worker DEG list resulted in substantial reductions in the number of genes (ovary list in current study reduced by 86% (2,827 to 408 genes), whole-body list in Harrison et al. (2015) reduced by 96% (1,208 to 44 genes)) (Table 1). Comparing the ovary-active worker DEG lists excluding egg-expressed genes still yielded significant overlap (7.6% of current study genes, *p* < 0.001) of DEGs from the current study and Harrison et al. (2015) (Fig. S5, Tables S11, S12).

In comparisons of enriched GO terms across fat body or ovary from the current study and Harrison et al. (2015), no overlap was present for ovary-active workers in ovary (0% of current study GO terms, *p* = 1) but significant overlaps were present for ovary-inactive workers in both fat body (28.1% of current study GO terms, *p* < 0.001) and ovary (20% of current study GO terms, *p* < 0.001) (Fig. S6, Tables S13, S14). Comparison was not possible using the ovary-active worker DEGs in fat body lists, or the ovary-active worker DEGs in ovary lists with egg-expressed genes excluded, as no GO terms were enriched in these gene lists for the current study (fat body) or Harrison et al. (2015) (ovary of ovary-active worker with egg-expressed genes excluded) (Fig. S6, Tables S13, S14). Overall, therefore, results from the current study and that of Harrison et al. (2015) were broadly congruent at the gene level, including for ovary with egg-expressed genes excluded, and in terms of GO enrichment.

**Comparison of gene expression differences associated with worker reproductivity in *Bombus* vs. *Apis***

Comparison of DEGs between *B. terrestris* and *A. mellifera* for ovary-active worker DEGs with egg-expressed genes included

Comparing DEGs from ovary in the current study to DEGs from *A. mellifera* ovary in Duncan et al. (2020) revealed no significant overlap in ovary-active worker DEGs with egg-expressed genes included (29.7% of current study genes, *p* = 0.047 (adjusted *p* value threshold for significance = 0.017), Fig. S1A**,** Tables S15, S16). Comparing DEGs from combined fat body and ovary in the current study to DEGs from *A. mellifera* combined fat body and ovary in Galbraith et al. (2016) revealed no significant overlap in ovary-active worker DEGs with egg-expressed genes included (18.5% of current study genes, *p* = 0.070, Fig. S1B**,** Tables S15, S16). For ovary-active worker DEGs with egg-expressed genes included, there were 336 overlapping DEGs between the current study and both *A. mellifera* studies (Table S19).

Among the DEGs expressed in both *B. terrestris* and *A. mellifera* eggs was a gene involved in reproduction in workers of the 'anarchist' *A. mellifera* lineage, *Anarchy* (Ronai et al. 2016) (*Bombus LOC100644134* and *Apis LOC551241*). The 'anarchy' syndrome in this *A. mellifera* lineage is characterised by high and unregulated levels of queenright worker egg-laying (Ronai et al. 2016). However, in worker ovary, *Anarchy* showed opposite patterns of differential expression between *B. terrestris* and *A. mellifera*, being more highly expressed in ovary of *B. terrestris* ovary-active workers than ovary-inactive ones but more highly expressed in ovary of *A. mellifera* ovary-inactive workers than ovary-active ones. The *A. mellifera* data are consistent with previous information regarding *Anarchy* in this species (Ronai et al. 2016). The *B. terrestris* data suggest that *Anarchy* does not play a corresponding role in *Bombus*, although it cannot be ruled out that the *B. terrestris* data stem from the expression of *Anarchy* in eggs.

Comparison of enriched GO terms between *B. terrestris* and *A. mellifera* for ovary-active worker DEGs with egg-expressed genes included

Comparing enriched GO terms from ovary in the current study to enriched GO terms from *A. mellifera* ovary in Duncan et al. (2020) revealed no significant overlap in GO terms enriched in ovary-active worker DEGs with egg-expressed genes included (0% of current study enriched GO terms, *p* = 1, Fig. S2A**,** Tables S20, S21). Comparing enriched GO terms from combined fat body and ovary in the current study to enriched GO terms from *A. mellifera* combined fat body and ovary in Galbraith et al. (2016) also revealed no significant overlap in GO terms enriched in ovary-active worker DEGs with egg-expressed genes included (0% of current study enriched GO terms, *p* = 1, Fig. S2B, Tables S22, S23).

**Behavioural-morphological caste homology (BMCH) hypothesis tests**

Comparison of DEGs between *B. terrestris* workers and larvae for ovary-active worker DEGs with egg-expressed genes included

As regards BMCH hypothesis prediction 1 at the gene level, the results showed significant absence of overlap between DEGs from ovary of ovary-active workers with egg-expressed genes included and queen-destined larvae in mid instars (*p* = 0.001, Fig. S3A), and no significant overlap in late instars (Fig. S3B) (Tables S24, S25).

Comparison of enriched GO terms between *B. terrestris* workers and larvae for ovary-active DEGs with egg-expressed genes included

As regards BMCH hypothesis prediction 1 at the GO level, comparing enriched GO terms derived from DEGs in the current study from ovary of ovary-active workers with egg-expressed genes included and queen-destined larvae showed no significant overlap for either mid or late instars (0% of current study enriched GO terms, *p* = 1 in both cases) (Fig. S4, Table S26).

**Summary of effects on comparisons of excluding or including egg-expressed genes**

The exclusion or inclusion of egg-expressed genes in comparisons using gene lists from ovary of workers potentially created differences in the outcomes of comparisons involving ovary of ovary-active workers only, because ovary of worker-inactive workers contains no eggs (see above, 'Egg sequencing experiment analysis'). To summarise these differences for the comparisons of gene expression between *Bombus* and *Apis*: the comparisons of ovary-active worker DEGs with egg-expressed genes excluded found significant overlap in 2/2 comparisons (main text, 'Comparison of DEGs between *B. terrestris* and *A. mellifera*'), whereas the comparisons of ovary-active worker DEGs with egg-expressed genes included found significant overlap in 0/2 comparisons (see above, 'Comparison of gene expression differences associated with worker reproductivity in *Bombus* vs. *Apis*'); comparisons of enriched GO terms from ovary-active worker DEGs with egg-expressed genes excluded were not possible (because of a lack of enriched GO terms in the two *A. mellifera* studies when egg-expressed genes were excluded) (main text, 'Comparison of Enriched GO Terms between *B. terrestris* and *A. mellifera*'), and the comparisons of enriched GO terms from ovary-active worker DEGs with egg-expressed genes included found significant overlap in 0/2 comparisons (see above, 'Comparison of gene expression differences associated with worker reproductivity in *Bombus* vs. *Apis*').

Therefore, the effect of excluding egg-expressed genes was to strengthen the support for overlaps between genes and gene pathways associated with worker reproductivity in *Bombus* vs. *Apis*. However, overall support for significant overlaps in this context between the taxa remained whether egg-expressed genes were excluded or not, because the comparisons of ovary-inactive worker DEGs found significant overlap in 1/2 comparisons (main text, 'Comparison of DEGs between *B. terrestris* and *A. mellifera*') and comparisons of enriched GO terms from ovary-inactive worker DEGs found significant overlap in 2/2 comparisons (main text, 'Comparison of Enriched GO Terms between *B. terrestris* and *A. mellifera*'), with (as above) both these sets of comparisons being unaffected by the exclusion or inclusion of egg-expressed genes.

Similarly, to summarise such differences for the tests of the BMCH hypothesis (with the exclusion or inclusion of egg-expressed genes affecting prediction 1 only, as prediction 2 involved comparing ovary-inactive workers to worker-destined larvae): as regards prediction 1 at the gene level, the results showed significant overlap between DEGs from ovary of ovary-active workers with egg-expressed genes excluded and queen-destined larvae in 2/2 comparisons (main text, 'Comparison of DEGs between *B. terrestris* Workers and Larvae'), whereas, comparing DEGs from ovary of ovary-active workers with egg-expressed genes included and queen-destined larvae, they showed significant non-overlap in 1/1 comparisons and significant overlap in 0/1 comparisons (see above, 'Behavioural-morphological caste homology (BMCH) hypothesis tests'); as regards prediction 1 at the GO level, testing for significant overlap in enriched GO terms derived from DEGs from ovary of ovary-active workers with egg-expressed genes excluded and queen-destined larvae was not possible, since DEGs with egg-expressed genes excluded for both instars of queen-destined larvae were not enriched for any GO terms (main text, 'Comparison of Enriched GO Terms between *B. terrestris* Workers and Larvae'), and the results showed significant overlap in enriched GO terms derived from DEGs from ovary of ovary-active workers with egg-expressed genes included and queen-destined larvae in 0/2 comparisons (see above, 'Behavioural-morphological caste homology (BMCH) hypothesis tests').

Therefore, as regards prediction 1 of the BMCH hypothesis, at the gene level the results with egg-expressed genes excluded provided support for the prediction, whereas results with egg-expressed genes included did not support it. Indeed the latter results were arguably counter to it by suggesting different DEGs underpin the ovary-active worker phenotype in worker ovary and the queen-destined pathway in mid-instar female larvae; however, this finding may have been less reliable because excluding egg-expressed genes resulted in the more conservative gene set (main text, 'Conclusions'). At the gene ontology level, including egg-expressed genes rendered tests possible and returned findings of no significant overlaps, which, although this outcome may again have been less reliable, was consistent with the findings of the tests of prediction 2 (unaffected by exclusion or inclusion of egg-expressed genes) at the gene ontology level that the BMCH hypothesis was not supported at this level (main text, 'Discussion, Behavioural-Morphological Caste Homology Hypothesis Tests').

**Supplementary References**

Ammar, R. and Thompson, J. 2021. zFPKM: A suite of functions to facilitate zFPKM transformations. <https://github.com/ronammar/zFPKM/>.

Andrews, S. 2015. FastQC: a quality control tool for high throughput sequence data., Babraham Bioinformatics.

Bray, N. L., Pimentel, H., Melsted, P. and Pachter, L. 2016. Near-optimal probabilistic RNA-seq quantification. *Nature Biotechnology* **34**(5):525-527 10.1038/nbt.3519.

Collins, D. H. et al. 2021. Gene expression during larval caste determination and differentiation in intermediately eusocial bumblebees, and a comparative analysis with advanced eusocial honeybees. *Molecular Ecology* **30**(3):718-735 <https://doi.org/10.1111/mec.15752>.

Duchateau, M. J., Velthuis, H. H. W. and Boomsma, J. J. 2004. Sex ratio variation in the bumblebee *Bombus terrestris*. *Behavioral Ecology* **15**(1):71-82 10.1093/beheco/arg087.

Duncan, E. J., Leask, M. P. and Dearden, P. K. 2020. Genome architecture facilitates phenotypic plasticity in the honeybee (*Apis mellifera*). *Molecular Biology and Evolution* **37**(7):1964-1978 10.1093/molbev/msaa057.

Galbraith, D. A. et al. 2016. Testing the kinship theory of intragenomic conflict in honey bees (*Apis mellifera*). *Proceedings of the National Academy of Sciences* **113**(4):1020-1025 10.1073/pnas.1516636113.

Harrison, M. C., Hammond, R. L. and Mallon, E. B. 2015. Reproductive workers show queenlike gene expression in an intermediately eusocial insect, the buff-tailed bumble bee *Bombus terrestris*. *Molecular Ecology* **24**(12):3043-3063 10.1111/mec.13215.

Hart, T., Komori, H. K., LaMere, S., Podshivalova, K. and Salomon, D. R. 2013. Finding the active genes in deep RNA-seq gene expression studies. *BMC Genomics* **14**(1):778 10.1186/1471-2164-14-778.

Kim, D., Langmead, B. and Salzberg, S. L. 2015. HISAT: a fast spliced aligner with low memory requirements. *Nature Methods* **12**(4):357-360 10.1038/nmeth.3317.

Love, M. I., Huber, W. and Anders, S. 2014. Moderated estimation of fold change and dispersion for RNA-seq data with DESeq2. *Genome Biology* **15**(12):550 10.1186/s13059-014-0550-8.

Pires, C. V., Freitas, F. C. d. P., Cristino, A. S., Dearden, P. K. and Simões, Z. L. P. 2016. Transcriptome analysis of honeybee (*Apis mellifera*) haploid and diploid embryos reveals early zygotic transcription during cleavage. *PLoS ONE* **11**(1):e0146447 10.1371/journal.pone.0146447.

R Core Team. 2018. R: A language and environment for statistical computing. Vienna, Austria, R Foundation for Statistical Computing.

Ronai, I. et al. 2016. *Anarchy* is a molecular signature of worker sterility in the honey bee. *Molecular Biology and Evolution* **33**(1):134-142 10.1093/molbev/msv202.

Sadd, B. M. et al. 2015. The genomes of two key bumblebee species with primitive eusocial organization. *Genome Biology* **16**(1):76 10.1186/s13059-015-0623-3.

Soneson, C., Love, M. and Robinson, M. 2016. Differential analyses for RNA-seq: transcript-level estimates improve gene-level inferences. *F1000Research* **4**([version 2; peer review: 2 approved].) <https://doi.org/10.12688/f1000research.7563.2>.

Wallberg, A. et al. 2019. A hybrid de novo genome assembly of the honeybee, *Apis mellifera*, with chromosome-length scaffolds. *BMC Genomics* **20**(1):275 10.1186/s12864-019-5642-0.

**Supplementary Figures**


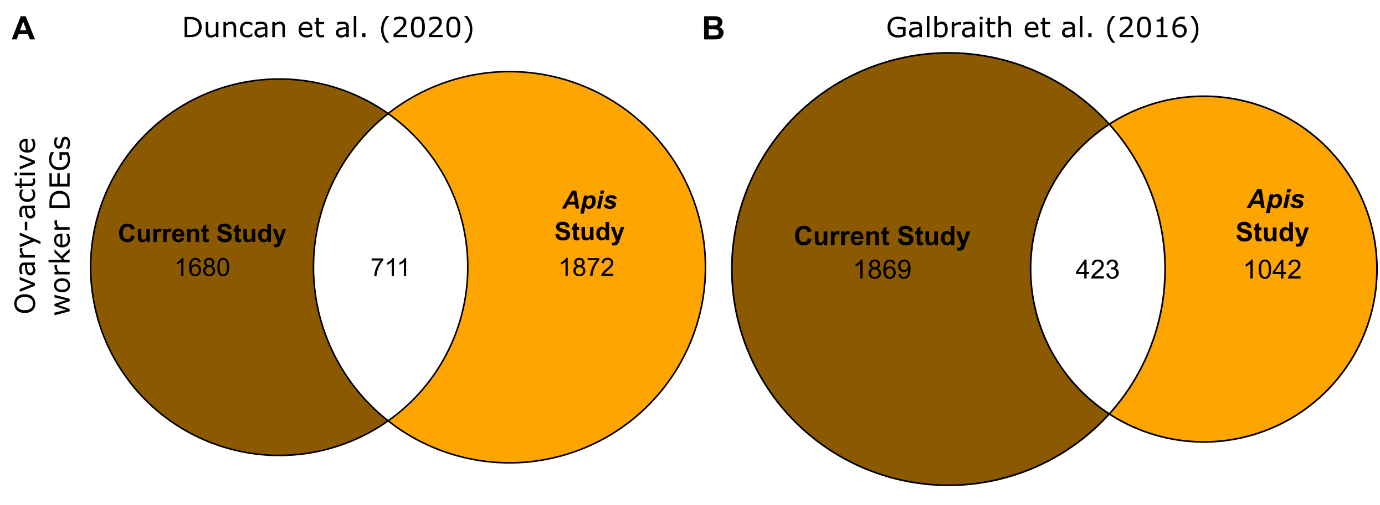


Figure S1. Comparison of gene expression in ovary of ovary-active workers with egg-expressed genes included between *Bombus terrestris* and *Apis mellifera* workers. Euler diagrams of overlaps in differentially expressed genes (DEGs) from mRNA-seq data for ovary-active vs. ovary-inactive workers between *B. terrestris* (current study) and *A. mellifera* (Duncan et al. (2020); Galbraith et al. (2016)) ('*Apis* study') for ovary-active worker DEGs in ovary with egg-expressed genes included and; **A,** Duncan et al. (2020); **B**, Galbraith et al. (2016). Numbers are number of DEGs in each category. Results of statistical tests are in Tables S15 and S17 and identities of DEGs are in Tables S16 and S18.


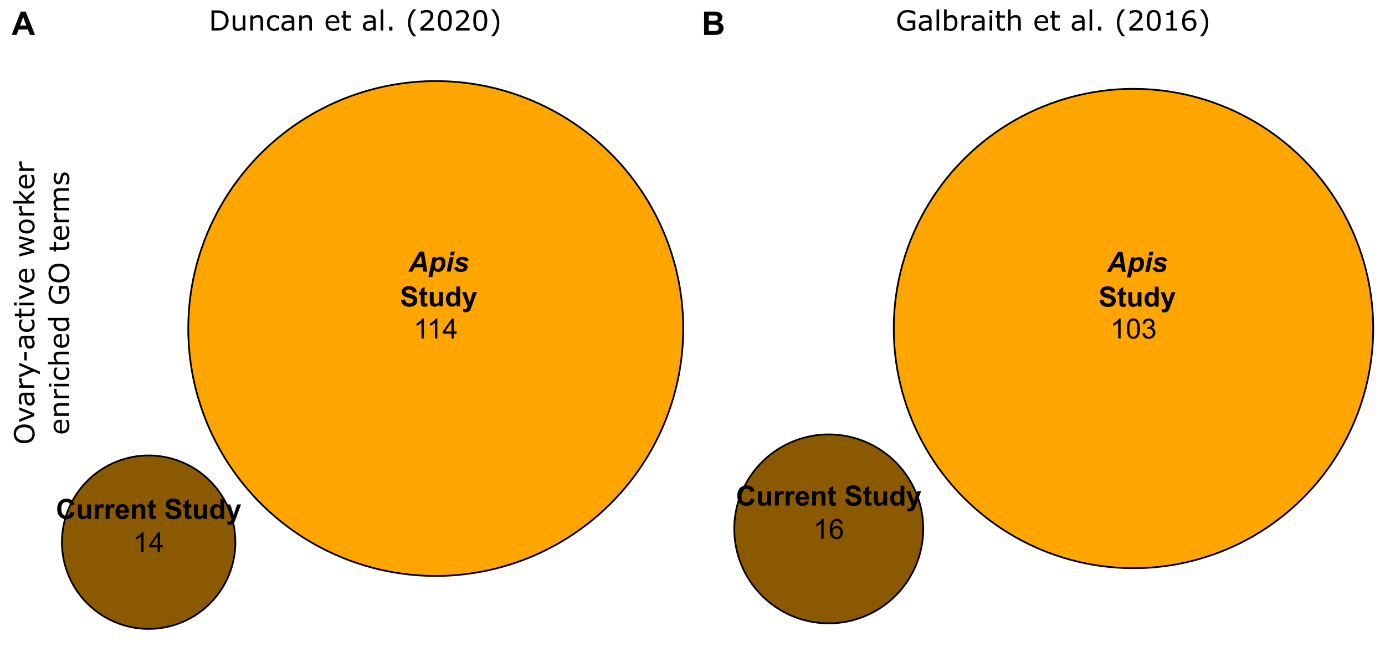


Figure S2. Comparison of gene ontology in ovary of ovary-active workers with egg-expressed genes included between *Bombus terrestris* and *Apis mellifera* workers. Euler diagrams of overlaps in enriched gene ontology (GO) terms derived from mRNA-seq data for ovary-active vs. ovary-inactive workers between *B. terrestris* (current study) and *A. mellifera* (Duncan et al. (2020); Galbraith et al. (2016)) ('*Apis* study') for ovary-active worker DEGs in ovary with egg-expressed genes included and; **A,** Duncan et al. (2020); **B**, Galbraith et al. (2016). Numbers are number of enriched GO terms in each category. Results of statistical tests are in Tables S20 and S22 and identities of GO terms are in Tables S21 and S23.


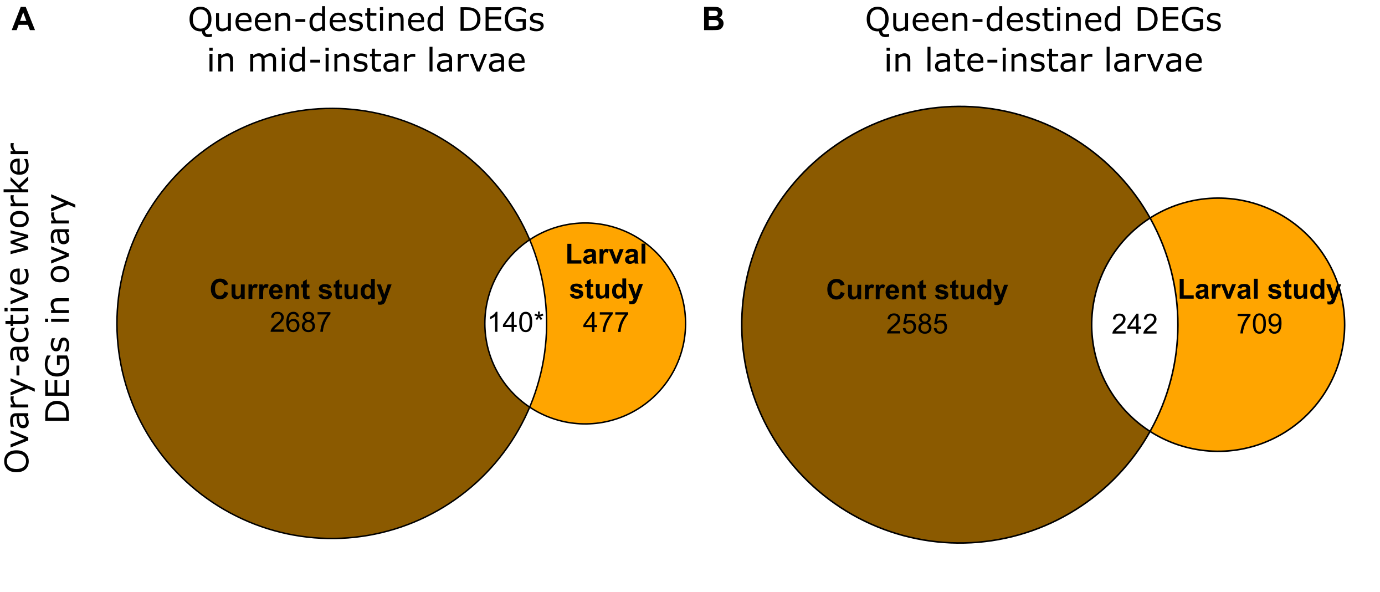


Figure S3. Comparison of gene expression in *Bombus terrestris* between ovary of ovary-active adult workers and queen-destined larvae with egg-expressed genes included (BMCH hypothesis prediction 1). Euler diagrams of overlaps between differentially expressed genes (DEGs) from mRNA-seq data in ovary (with egg-expressed genes included) in ovary-active vs. ovary-inactive *B. terrestris* workers (current study) and in mid and late-instars of queen-destined vs. worker-destined *B. terrestris* whole larvae (Collins et al. 2021) ('Larval study') for: **A**, mid-instar larvae; and **B**, late-instar larvae. Numbers are number of DEGs in each category. Asterisks (*), significant absence of overlap in DEGs (Fisher’s exact test, *p* < 0.05 after Bonferroni correction (adjusted *p* value threshold for significance = 0.0083)) Results of statistical tests are in Table S24.


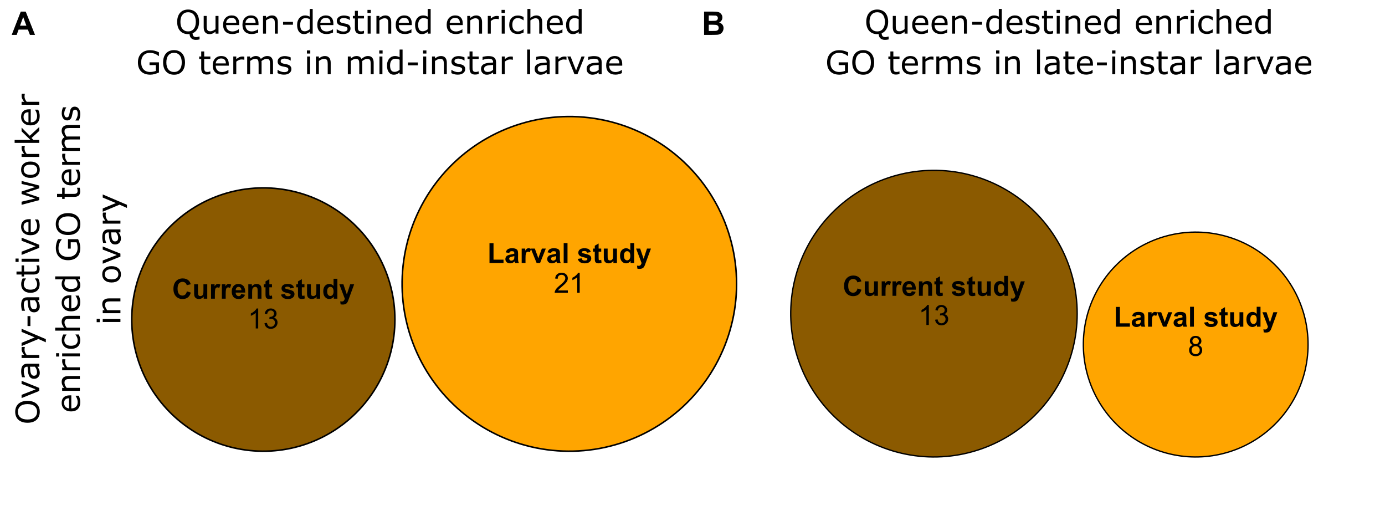


Figure S4. Comparison of gene ontology in *Bombus terrestris* between ovary of ovary-active adult workers and queen-destined larvae with egg-expressed genes included (BMCH hypothesis prediction 1). Euler diagrams of overlaps between enriched gene ontology (GO) terms from mRNA-seq data in ovary (with egg-expressed genes included) in ovary-active vs. ovary-inactive *B. terrestris* workers (current study) and in mid and late-instars of queen-destined vs. worker-destined *B. terrestris* whole larvae (Collins et al. 2021) ('Larval study') for : **A**, mid-instar larvae; and **B**, late-instar larvae. Numbers are number of DEGs in each category. Results of statistical tests are in Table S26.


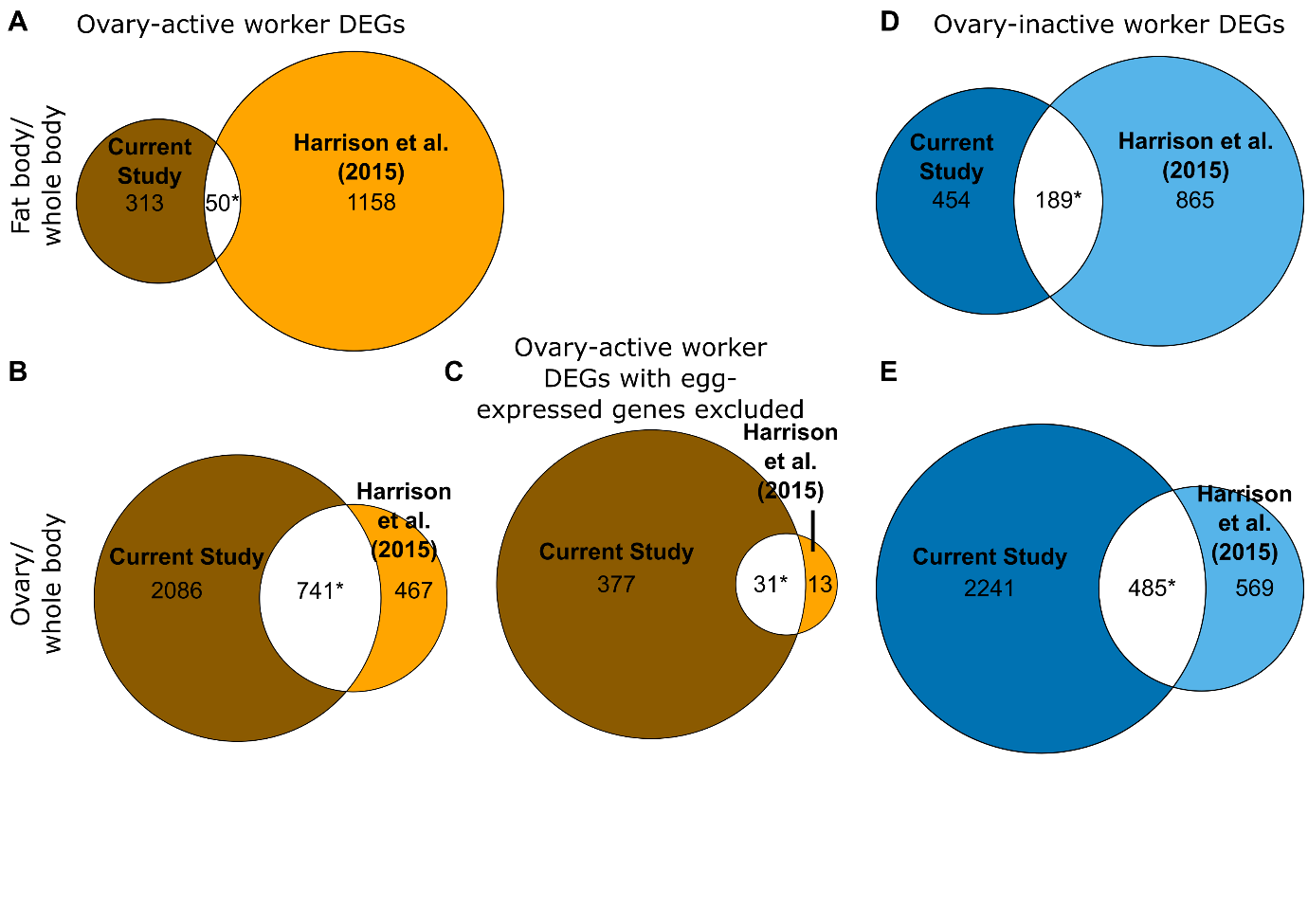


Figure S5. Overlap in genes differentially expressed between fat body or ovary of ovary-active and ovary-inactive *Bombus terrestris* workers in current study and whole bodies of ovary-active and ovary-inactive *B. terrestris* workers from Harrison et al. (2015). Euler diagrams of overlaps between differentially expressed genes (DEGs) for: **A, B**, ovary-active worker DEGs with egg-expressed genes included; **C**, ovary-active worker DEGs with egg-expressed genes excluded; and **D, E**, ovary-inactive worker DEGs. A, D, fat body from the current study vs. whole body from Harrison et al. (2015); B, C, E, ovary from the current study vs. whole body from Harrison et al. (2015). Numbers are number of DEGs in each category. Asterisks (*), significant overlap in DEGs (Fisher's exact test, *p* < 0.05 after Bonferroni correction (adjusted *p* value threshold for significance = 0.01)). Results of statistical tests are in Table S11 and identities of overlapping genes are in Table S12.


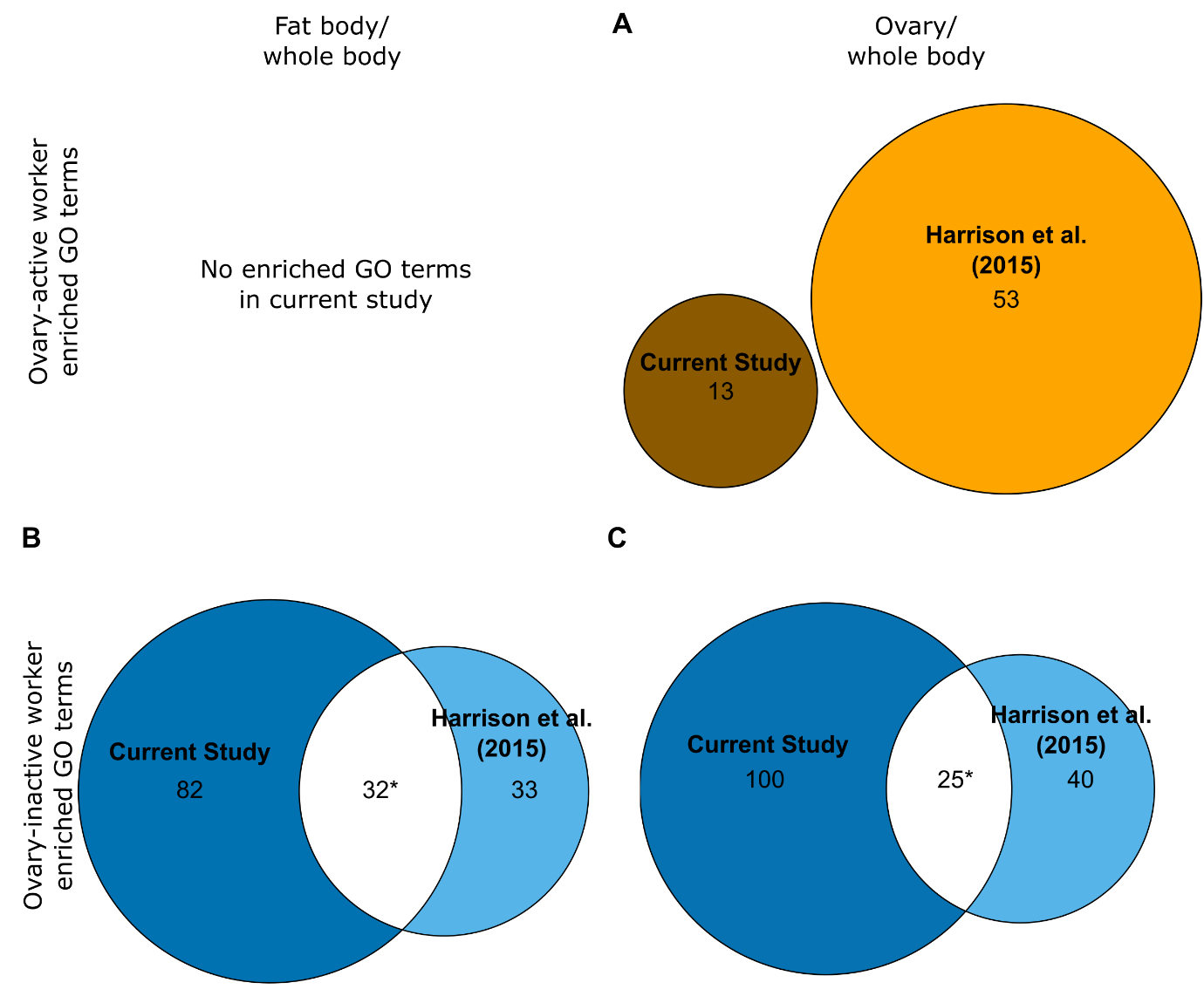


Figure S6. Overlap in enriched gene ontology terms between fat body or ovary of ovary-active and ovary-inactive *Bombus terrestris* workers in current study and whole bodies of ovary-active and ovary-inactive *B. terrestris* workers from Harrison et al. (2015). Euler diagrams of overlaps between enriched gene ontology (GO) terms for: **A**, ovary-active worker DEGs in ovary of the current study vs. whole body from Harrison et al. (2015); **B**, ovary-inactive worker DEGs in fat body of the current study vs. whole body from Harrison et al. (2015); and **C**, ovary-inactive worker DEGs in ovary of the current study vs. whole body from Harrison et al. (2015). Numbers are number of enriched GO terms in each category. Asterisks (*), significant overlap in enriched GO terms (Fisher's exact test, *p* < 0.05 after Bonferroni correction (adjusted *p* value threshold for significance = 0.01)). Results of statistical tests are in Table S13 and identities of overlapping enriched GO terms are in Table S14.


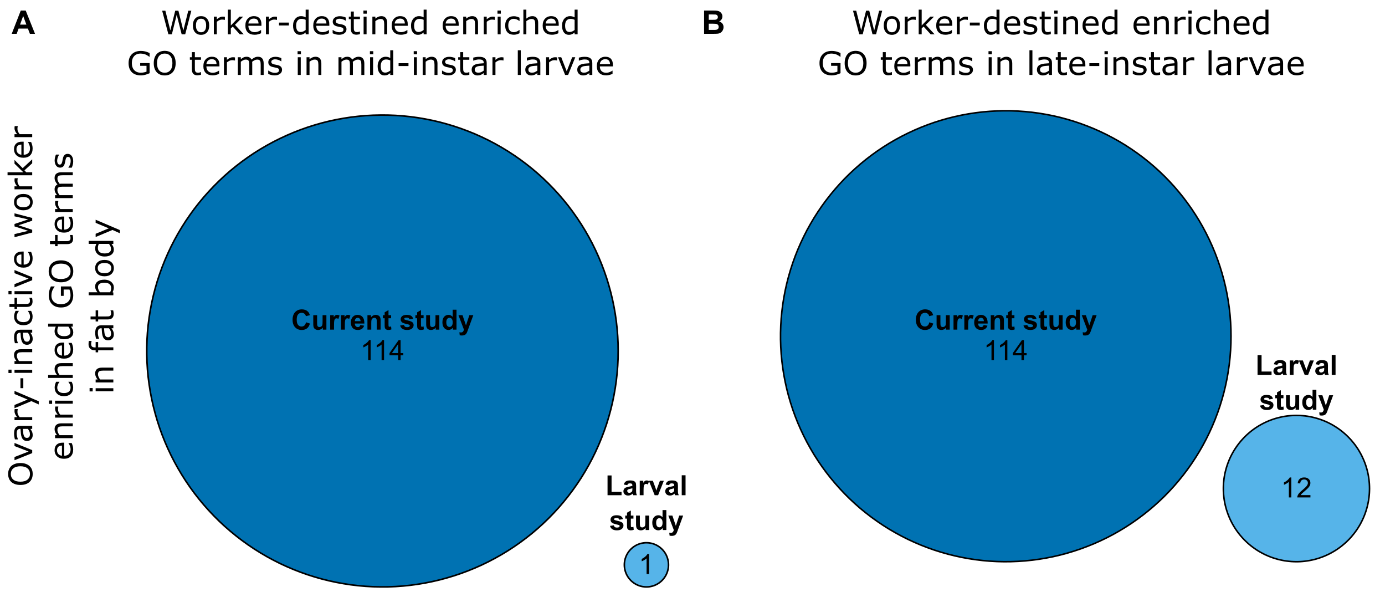


Figure S7. Comparison of enriched gene ontology terms in fat body of *Bombus terrestris* ovary-inactive adult workers and worker-destined larvae (BMCH hypothesis prediction 2). Euler diagrams of overlaps between enriched gene ontology (GO) terms from mRNA-seq data in fat body in ovary-active vs. ovary-inactive *B. terrestris* workers (current study) and: **A**, mid-instar larvae; and **B**, late-instar larvae from Collins et al. 2021 ('Larval study'). Numbers are number of enriched GO terms in each category. Results of statistical tests are in Table S26.


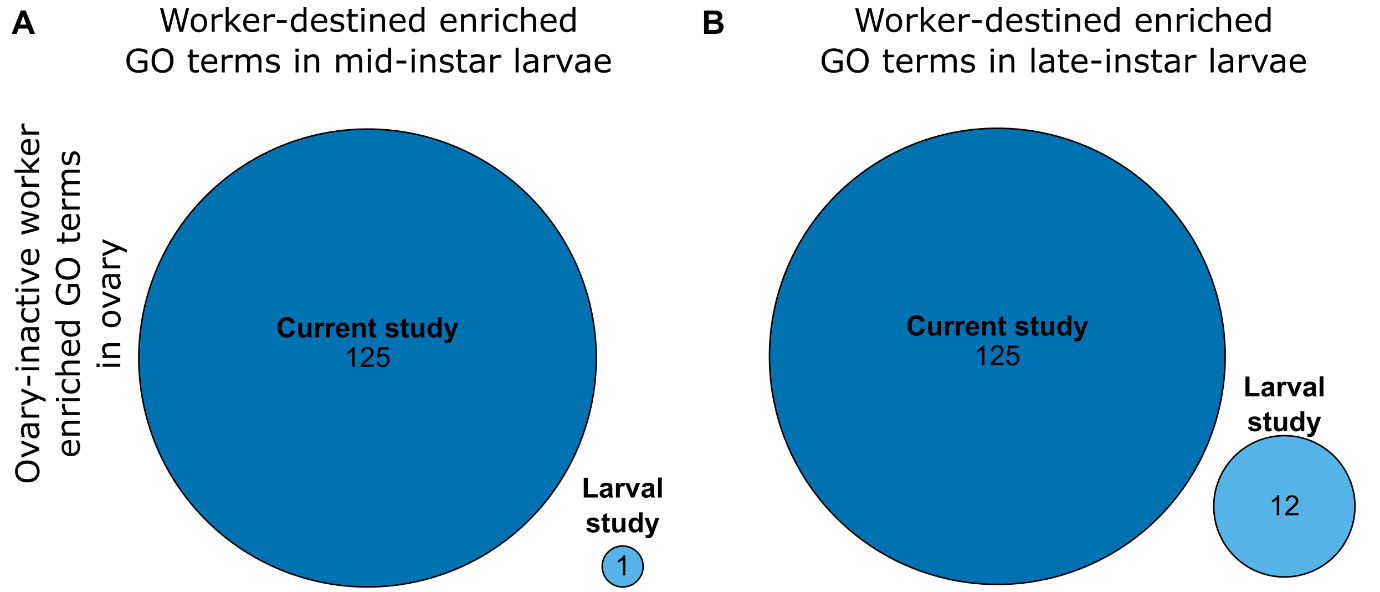


Figure S8. Comparison of enriched gene ontology terms in ovary of *Bombus terrestris* ovary-inactive adult workers and worker-destined larvae (BMCH hypothesis prediction 2). Euler diagrams of overlaps between enriched gene ontology (GO) terms from mRNA-seq data in ovary in ovary-active vs. ovary-inactive *B. terrestris* workers (current study) and: **A**, mid-instar larvae; and **B**, late-instar larvae from Collins et al. 2021 ('Larval study'). Numbers are number of enriched GO terms in each category. Results of statistical tests are in Table S26.


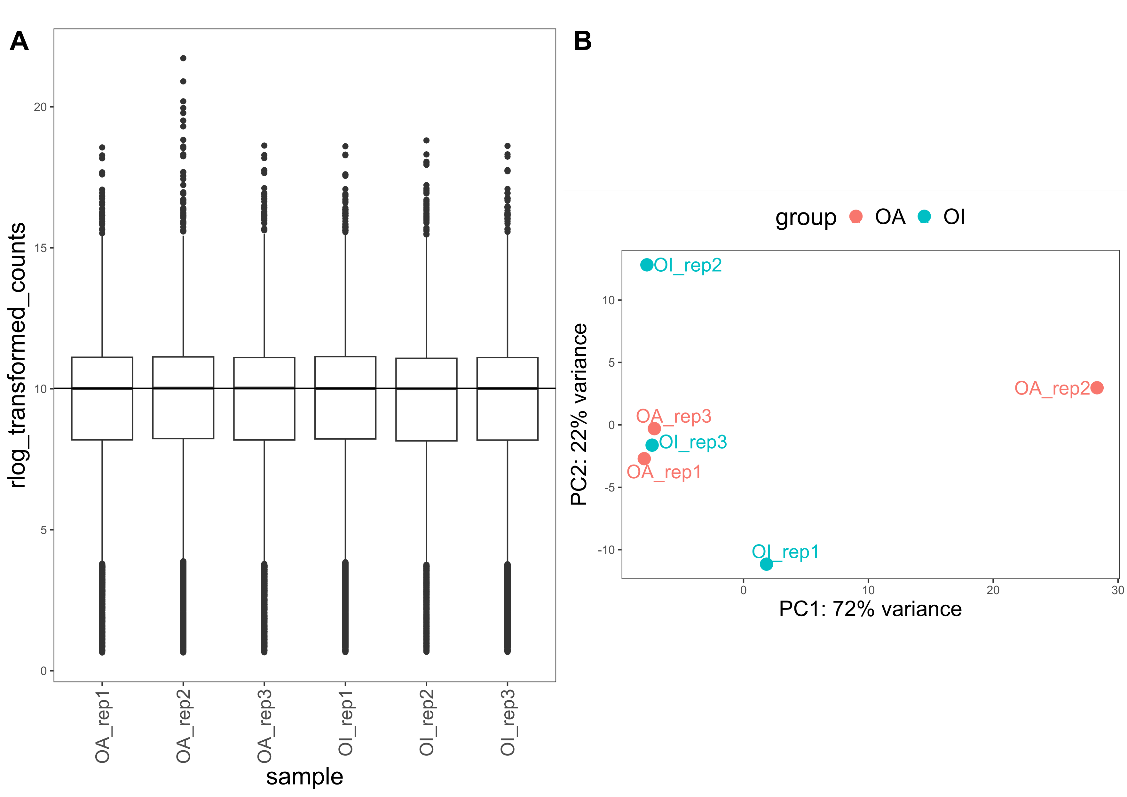


Figure S9. Exploratory plots from the differential gene expression analysis in pooled brain of *Bombus terrestris* ovary-active and ovary-inactive workers. **A**, Normalisation boxplots of the regularised log_2_- (rlog) transformed value of mRNA-seq expression for genes in all libraries. Black horizontal bars: medians; boxes: interquartile ranges; whiskers: 10th to 90th percentile ranges. **B**, Principal component analysis (PCA) plot of the top 2,000 most highly expressed genes isolated from all mRNA-seq libraries in brain; Axes represent principal components. Individual points represent biological replicates (coloured by group). A, B,, Library and group names are in the format, phenotype_biological replicate. OA, ovary-active workers; OI, ovary-inactive workers; rep1, biological replicate 1. Brain mRNA-seq libraries: N = 6.

**
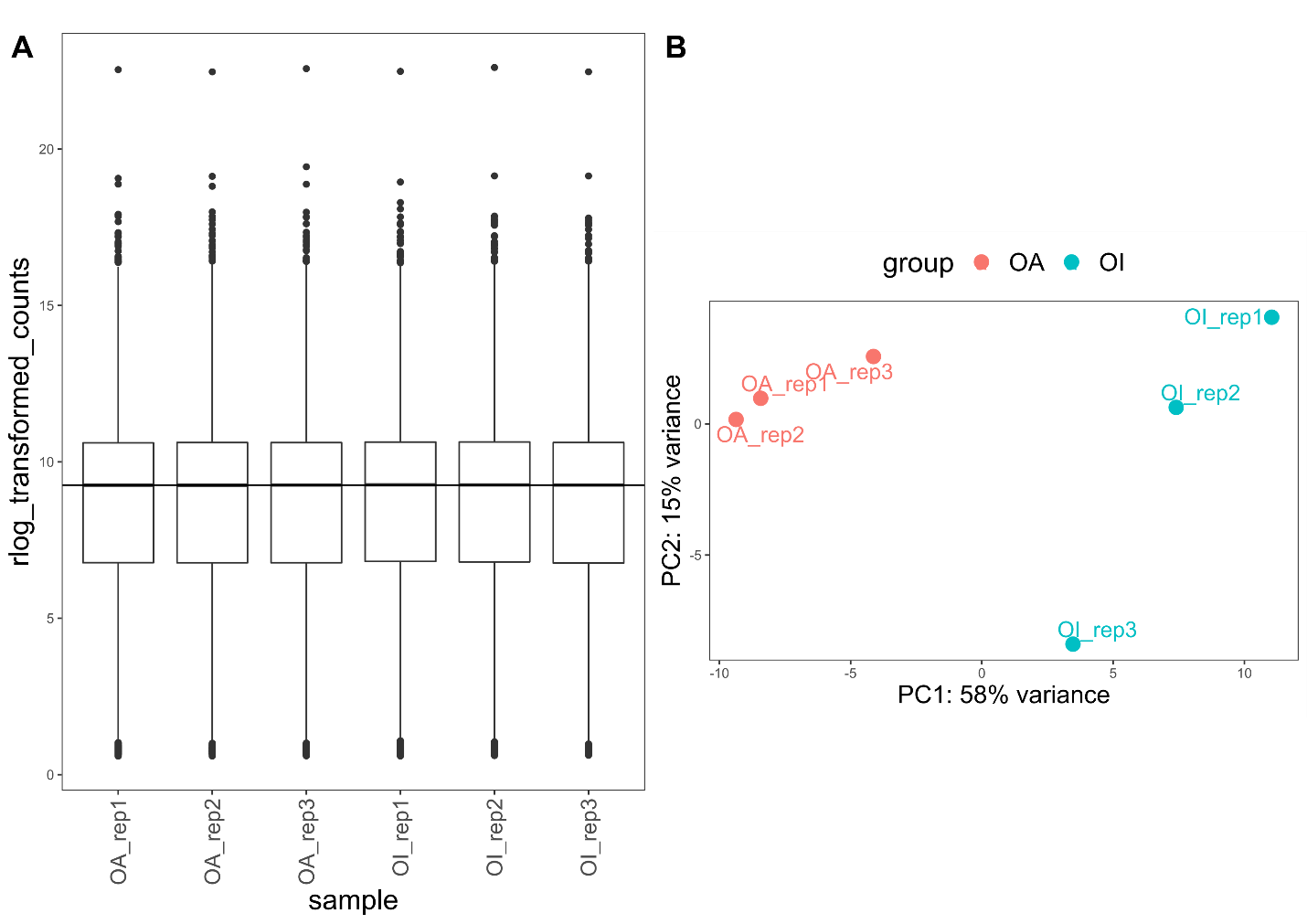
**

Figure S10. Exploratory plots from the differential gene expression analysis in pooled fat body of *Bombus terrestris* ovary-active and ovary-inactive workers. **A**, Normalisation boxplots of the regularised log_2_- (rlog) transformed value of mRNA-seq expression for genes in all libraries. Black horizontal bars: medians; boxes: interquartile ranges; whiskers: 10th to 90th percentile ranges. **B**, Principal component analysis (PCA) plot of the top 2,000 most highly expressed genes isolated from all mRNA-seq libraries in fat body. Axes represent principal components. Individual points represent biological replicates (coloured by group). Libraries are labelled with the library name. A, B, Library and group names are in the format, phenotype_biological replicate. OA, ovary-active workers; OI, ovary-inactive workers; rep1, biological replicate 1. Fat body mRNA-seq libraries: N = 6.


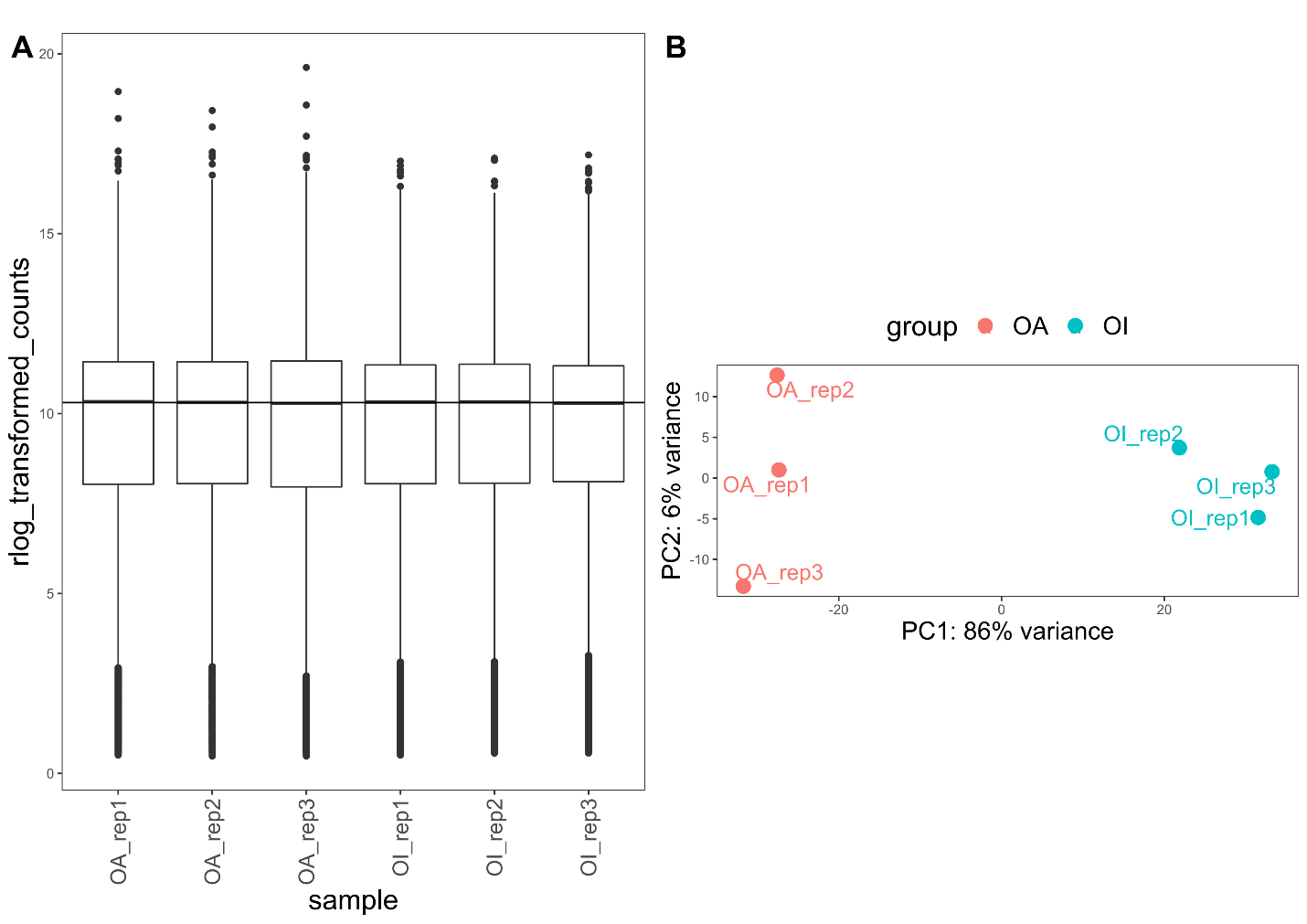


Figure S11. Exploratory plots from the differential gene expression analysis in pooled ovary of *Bombus terrestris* ovary-active and ovary-inactive workers. **A**, Normalisation boxplots of the regularised log_2_- (rlog) transformed value of mRNA-seq expression for genes in all libraries. Black horizontal bars: medians; boxes: interquartile ranges; whiskers: 10th to 90th percentile ranges. **B**, Principal component analysis (PCA) plot of the top 2,000 most highly expressed genes isolated from all mRNA-seq libraries in ovary. Axes represent principal components. Individual points represent biological replicates (coloured by group). Libraries are labelled with the library name. A, B, Library and group names are in the format, phenotype_biological replicate. OA, ovary-active workers; OI, ovary-inactive workers; rep1, biological replicate 1. Ovary mRNA-seq libraries: N = 6.


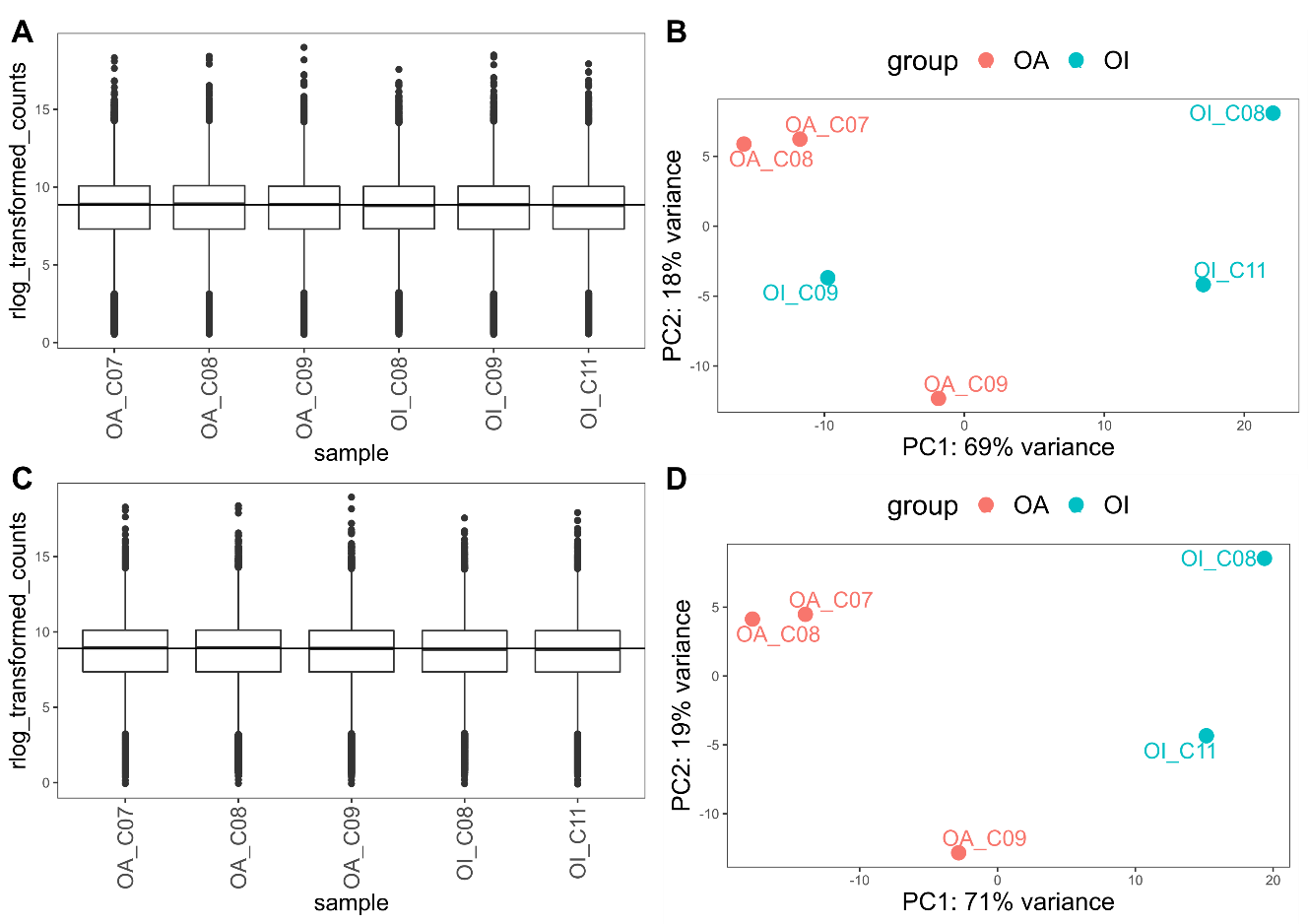


Figure S12. Exploratory plots from the differential gene expression analysis in whole bodies of *Bombus terrestris* ovary-active and ovary-inactive workers from Harrison et al. (2015). **A**, Normalisation boxplots of the regularised log_2_- (rlog) transformed value of mRNA-seq expression for genes in all libraries; **B**, Principal component analysis (PCA) plot of the top 2,000 most highly expressed genes isolated from all mRNA-seq libraries; **C**, Normalisation boxplots of the rlog-transformed value of mRNA-seq expression for genes in libraries with OI_C09 excluded; **D**, PCA plot of the top 2,000 most highly expressed genes isolated from mRNA-seq libraries with OI_C09 excluded. A, C, Black horizontal bars: medians; boxes: interquartile ranges; whiskers: 10th to 90th percentile ranges. B, D, Axes represent principal components. Individual points represent biological replicates (coloured by group). Libraries are labelled with the library name. A-D, Library and group names are in the format, phenotype_colony number. OA, ovary-active workers; OI, ovary-inactive workers; C09, colony 09. Harrison et al. (2015) mRNA-seq libraries: A, B; N = 6, C, D; N = 5.


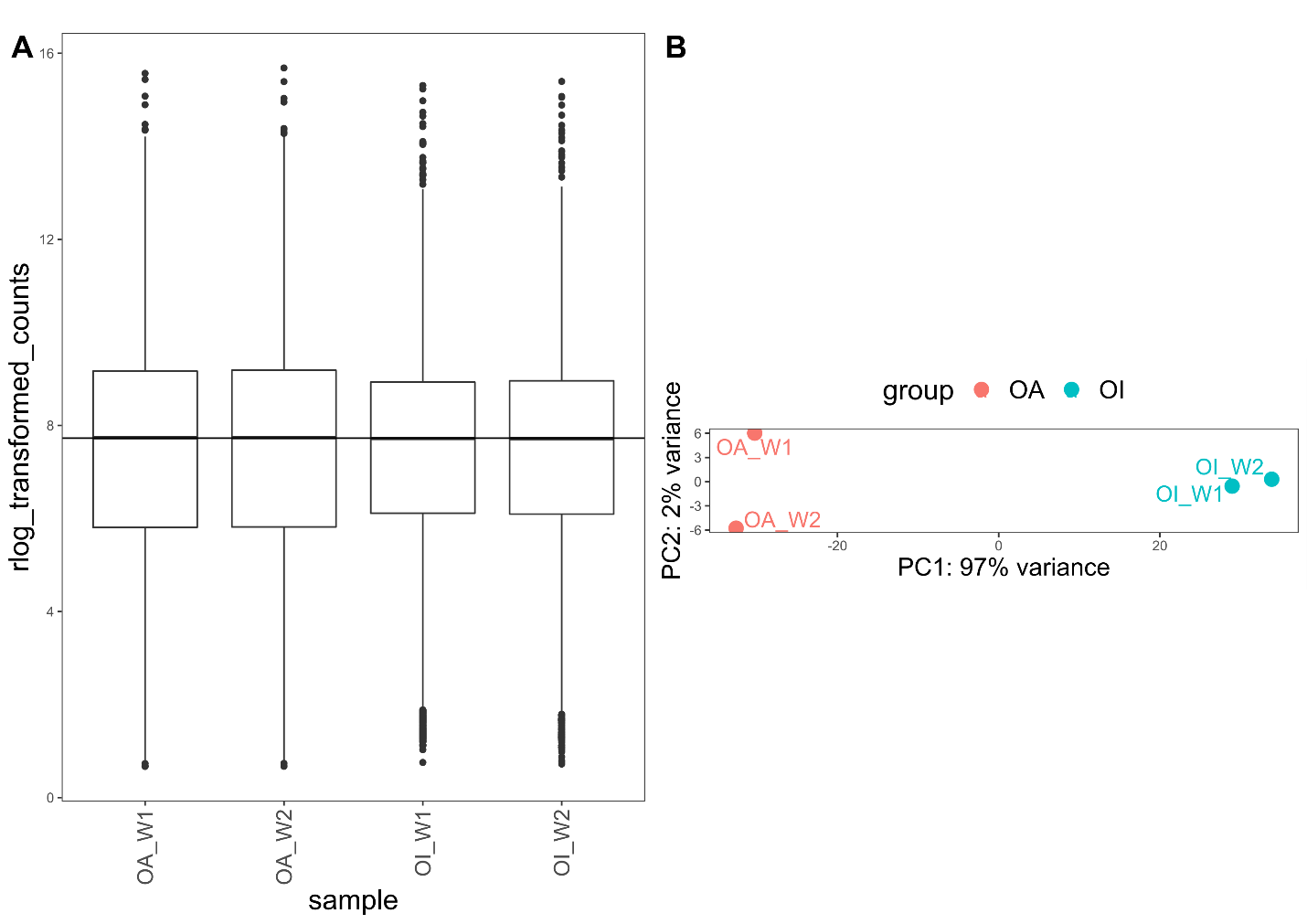


Figure S13. Exploratory plots from the differential gene expression analysis in ovary of *Apis mellifera* ovary-active and ovary-inactive workers from Duncan et al. (2020). **A**, Normalisation boxplots of the regularised log_2_- (rlog) transformed value of mRNA-seq expression for genes in all libraries. Black horizontal bars: medians; boxes: interquartile ranges; whiskers: 10th to 90th percentile ranges. **B**, Principal component analysis (PCA) plot of the top 2,000 most highly expressed genes isolated from all mRNA-seq libraries in Duncan et al. (2020). Axes represent principal components. Individual points represent biological replicates (coloured by group). Libraries are labelled with the library name. A, B, Library and group names are in the format, phenotype_biological replicate. OA, ovary-active workers; OI, ovary-inactive workers; W1, worker biological replicate 1. Duncan et al. (2020) mRNA-seq libraries: N = 4.


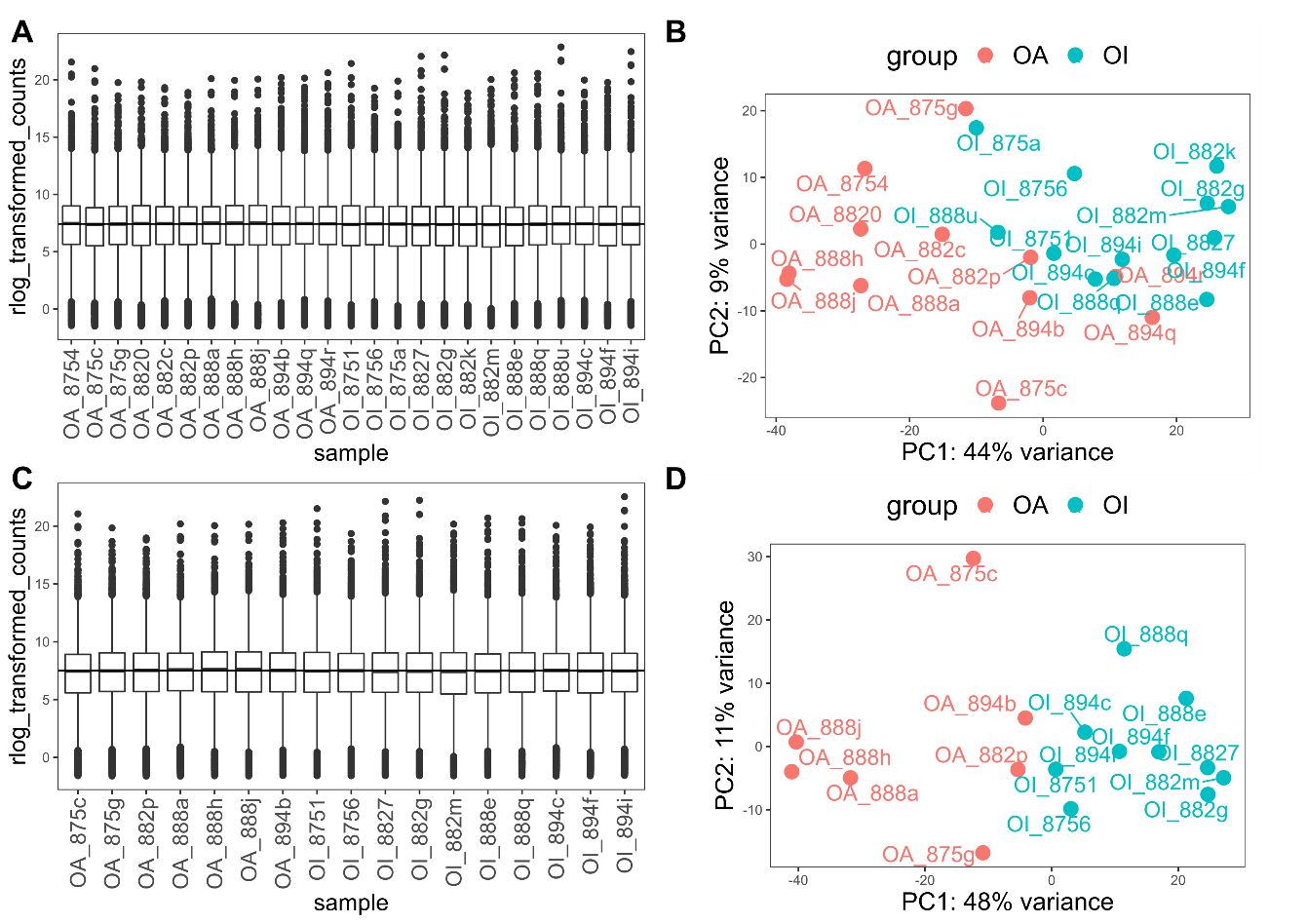


Figure S14. Exploratory plots from the differential gene expression analysis in combined fat body and ovary of *Apis mellifera* ovary-active and ovary-inactive workers from Galbraith et al. (2016). **A**, Normalisation boxplots of the regularised log_2_- (rlog) transformed value of mRNA-seq expression for genes in all libraries; **B**, Principal component analysis (PCA) plot of the top 2,000 most highly expressed genes isolated from all mRNA-seq libraries; **C**, Normalisation boxplots of the rlog-transformed value of mRNA-seq expression for genes with 8 libraries excluded; **D**, PCA plot of the top 2,000 most highly expressed genes isolated from mRNA-seq libraries with 8 libraries excluded. A, C, Black horizontal bars: medians; boxes: interquartile ranges; whiskers: 10th to 90th percentile ranges. B, D, Axes represent principal components. Individual points represent biological replicates (coloured by group). Libraries are labelled with the library name. A-D, Library and group names are in the format, phenotype_biological replicate. OA, ovary-active workers; OI, ovary-inactive workers; Galbraith et al. (2016) mRNA-seq libraries: A, B; N = 25, C, D; N = 17.


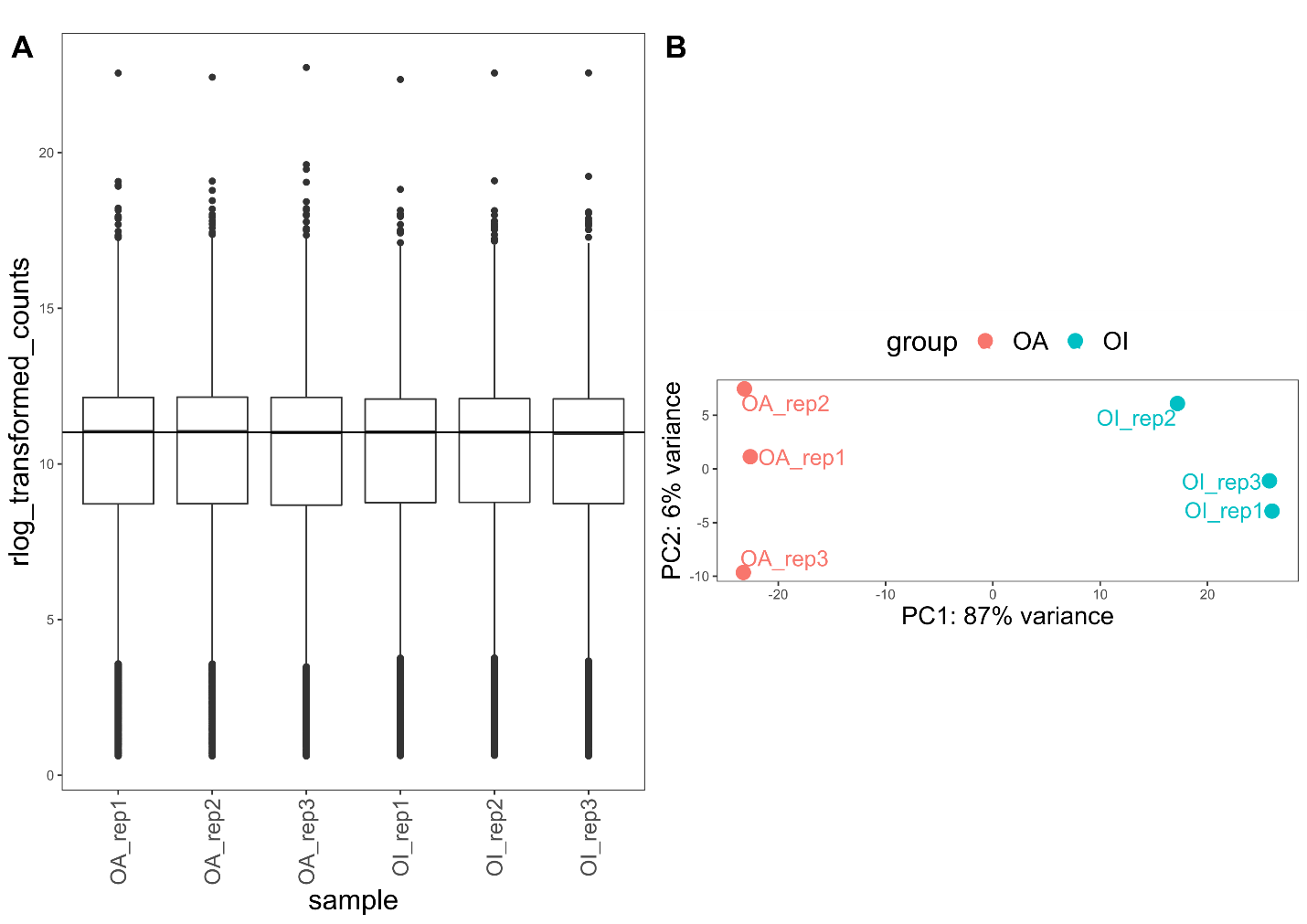


Figure S15. Exploratory plots from the differential gene expression analysis in combined fat body and ovary of *Bombus terrestris* ovary-active and ovary-inactive workers. **A**, Normalisation boxplots of the regularised log_2_- (rlog) transformed value of mRNA-seq expression for genes in all libraries. Black horizontal bars: medians; boxes: interquartile ranges; whiskers: 10th to 90th percentile ranges. **B**, Principal component analysis (PCA) plot of the top 2,000 most highly expressed genes isolated from all mRNA-seq libraries in combined fat body and ovary. Axes represent principal components. Individual points represent biological replicates (coloured by group). Libraries are labelled with the library name. A, B, Library and group names are in the format, phenotype_biological replicate. OA, ovary-active workers; OI, ovary-inactive workers; rep1, biological replicate 1. Combined fat body and ovary mRNA-seq libraries: N = 6.


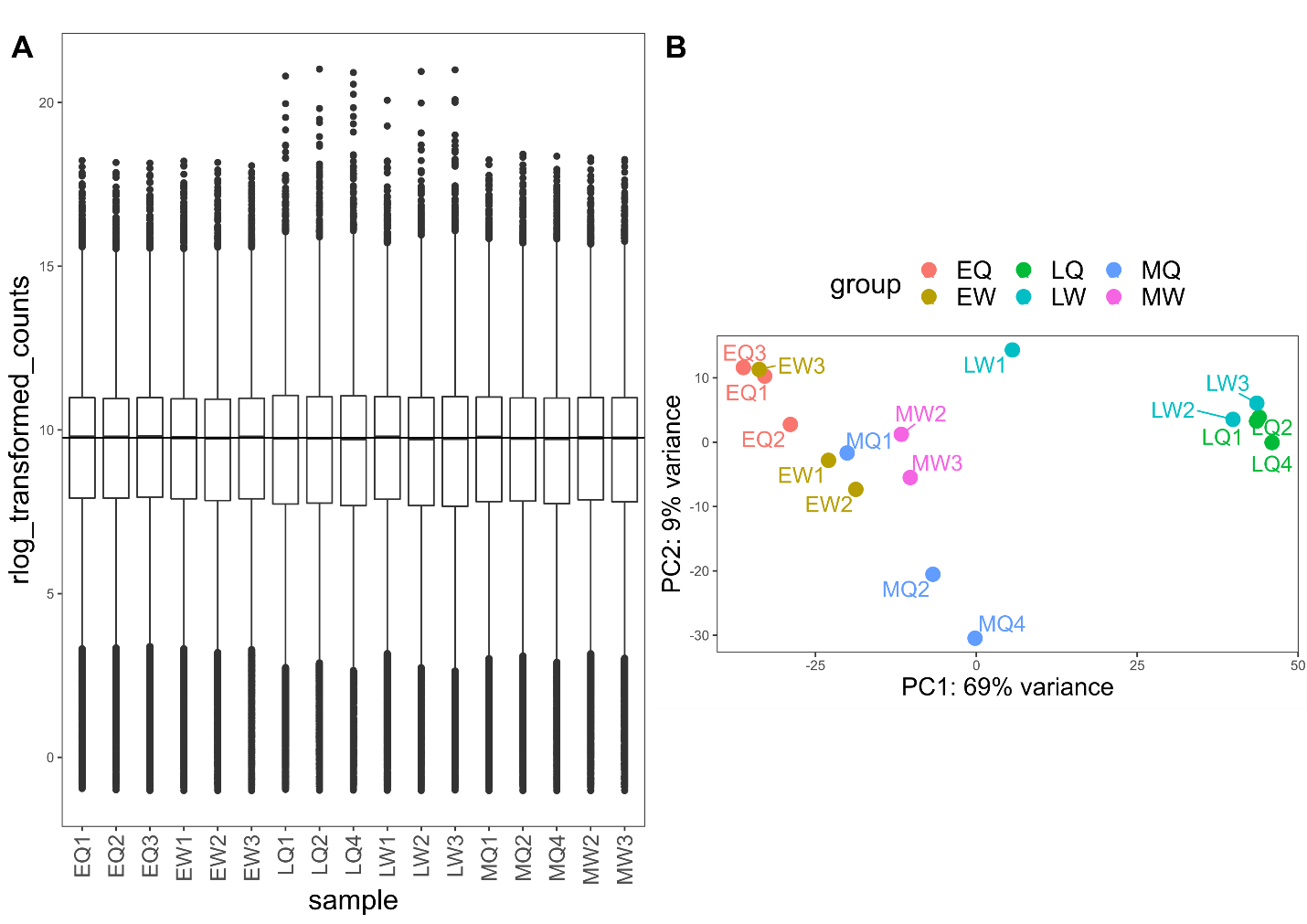


Figure S16. Exploratory plots from the differential gene expression analysis in pooled larvae of *Bombus terrestris* queen- and worker-destined larvae from Collins et al. (2021). **A**, Normalisation boxplots of the regularised log_2_- (rlog) transformed value of mRNA-seq expression for genes in all libraries. Black horizontal bars: medians; boxes: interquartile ranges; whiskers: 10th to 90th percentile ranges. **B**, Principal component analysis (PCA) plot of the top 2,000 most highly expressed genes isolated from all mRNA-seq libraries in queen- and worker-destined larvae. Axes represent principal components. Individual points represent biological replicates (coloured by group). Libraries are labelled with the library name. A, B, Library and group names are in the format, phenotype, biological replicate. E, early-instar larvae; M, mid-instar larvae; L, late-instar larvae; Q, queen-destined larvae; W, worker-destined larvae; 1, biological replicate 1. Larvae mRNA-seq libraries: N = 17.
